# Epigenetic regulation in cardiovascular disease: mechanisms and advances in clinical trials

**DOI:** 10.1038/s41392-022-01055-2

**Published:** 2022-06-25

**Authors:** Yuncong Shi, Huanji Zhang, Suli Huang, Li Yin, Feng Wang, Pei Luo, Hui Huang

**Affiliations:** 1 The Eighth Affiliated Hospital, Sun Yat-sen University, No. 3025, Shennan Middle Rd, Futian District, 518033 Shenzhen, China; 2 Shenzhen Center for Disease Control and Prevention, 518055 Shenzhen, China; 3 State Key Laboratory of Chinese Materia Medica Quality Research, Macau University of Science and Technology, 999078 Macao, China

**Keywords:** Cardiology, Molecular medicine

## Abstract

Epigenetics is closely related to cardiovascular diseases. Genome-wide linkage and association analyses and candidate gene approaches illustrate the multigenic complexity of cardiovascular disease. Several epigenetic mechanisms, such as DNA methylation, histone modification, and noncoding RNA, which are of importance for cardiovascular disease development and regression. Targeting epigenetic key enzymes, especially the DNA methyltransferases, histone methyltransferases, histone acetylases, histone deacetylases and their regulated target genes, could represent an attractive new route for the diagnosis and treatment of cardiovascular diseases. Herein, we summarize the knowledge on epigenetic history and essential regulatory mechanisms in cardiovascular diseases. Furthermore, we discuss the preclinical studies and drugs that are targeted these epigenetic key enzymes for cardiovascular diseases therapy. Finally, we conclude the clinical trials that are going to target some of these processes.

## Introduction

Cardiovascular diseases remain a major cause of destruction of human health worldwide.^1^ In the worldwide, especially China and India have the highest burden of cardiovascular disease.^2^ It includes coronary heart disease, hypertension, heart failure, vascular calcification and so on. In the Framingham Heart Study,^3^ which was launched since 1948 covering three generations during the past 70 years, cardiovascular disease is well studied and highly related to multiple factors such as biochemical, environmental, behavioral and genetics factors. Early detection and diagnosis of cardiovascular diseases are essential to improve the treatment and prognosis of cardiovascular disease.

With the rapid development of modern society, the incidence of cardiovascular diseases shows an increasing trend year by year, and the onset age is gradually younger. The causes of cardiovascular diseases such as coronary heart disease, heart failure, and hypertension are closely related to environmental factors and genetic factors. Recent studies have found that epigenetic modification plays an important role in the occurrence and development of cardiovascular diseases. Epigenetics is a regulation mechanism that can perpetuate alternative gene function/expression/activity without changing the content of DNA sequence. It is considered as the major response regulation mechanism for the cell response to the environmental changes.^4^ Epigenetics mainly regulates cardiovascular disease-related genes function and expression level through DNA methylation, histone modification, and noncoding RNA regulation, thus affecting cardiovascular disease progression. Epigenetic markers are important molecular markers of cardiovascular disease because they occur early in the disease and involve key cardiovascular pathologically related pathways. Most importantly, it can be used as cardiovascular disease biomarkers for cardiovascular disease diagnosis, treatment response prediction and evaluation. As we all know, the pathogenesis of cardiovascular disease remains intricate and complex. Clinically, some cases are still difficult to cure, and the prevalence rate increases with age. Interestingly, because of the reversibility of epigenetic modifications, genes and proteins that control these changes have become new targets for cardiovascular disease treatment. Therefore, new therapeutic strategies based on epigenetic modification have aroused great interest. The development of effective therapies for cardiovascular diseases is an urgent clinical need.

In this review, we illustrate (1) the epigenetics history and general mechanisms, (2) epigenetic regulatory mechanisms in cardiovascular disease, (3) epigenetics as a potential strategy for treating cardiovascular disease. The epigenetics in cardiovascular disease is yet to be cleared. There will be more comprehensive and large-scale epigenetics studies published in future. The novel epigenetics regulation mechanisms will provide additional opportunities to more promising cardiovascular disease diagnostic markers and potential therapeutic solutions. It is hoped that continued research in this area will further enhance the understanding of the cardiovascular disease process, which in order to get a better discovery of the new therapeutic strategy and improve the life quality of cardiovascular disease patients.

## Overview of epigenetics

### Epigenetics history brief review

The English biologist C.H. Waddington first proposed the term “epigenetics” in 1942, which ostensibly means “changes in non-genetic sequences.^5^ It was not until the 1980s that the British molecular biologist R Holliday systematically reformulated “Epigenetic” systematically in an academic paper according to the consensus that DNA methylation can change gene activity.^6^ In 1990, Holliday defined epigenetics as the study of the temporal and spatial mechanisms subjects by which genes activity during the development of complex organisms.^7^ In 1996, American geneticist Athur D. Riggs and others defined epigenetics as the genetic changes caused by mitosis or meiosis in the function of genes without changing the genetic sequence.^8,9^ In 2007, the British geneticist S A Bird defined epigenetics as the structural adjustment of the chromosomal region that causes it to express, emit signal, or maintain altered activity state.^10^ In 2008, at a cold Spring Harbor conference, the nature of epigenetics was recognized as chromosomal changes that caused stable inherited phenotype in the absence of DNA order changes. In addition, according to the extension of epigenetics research, the United States NATIONAL Institutes of Health (NIH) in 2013 believed that epigenetics included both cells or individuals gene activity and expression inherited changes, and stable, long-term, and uninherited changes at the potential level of cell transcription. At present, the widely accepted concept of epigenetics is the study that not DNA sequence changes caused heritable gene expression changes.^11^ The epigenetic mechanisms are discovered and widely accepted because it regulates gene expression without changing DNA sequence by covalent modifications made to histone proteins and nucleic acids that cooperatively regulate chromatin structure. The epigenetic regulations are reversible and dynamically regulated gene expression. It indicates that epigenetic mechanisms might play more important roles in biology. It also opens the possibility to develop epigenetic drugs for certain diseases. Taken together, the above section is a brief review of epigenetics history. Epigenetics mechanisms will be described below.

## Epigenetic regulatory mechanisms

### DNA methylation

**Fig. 1:**
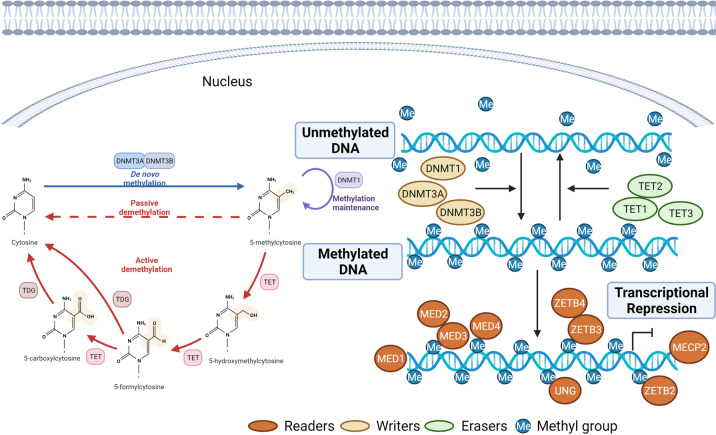
DNA methylation regulation. DNA methylation occurs mainly in the islands of cytosinephosphateguanine (CpG) gene promoter region. It promotes gene transcription in the promoter region by activating DNA methyltransferases. DNA methylases can be divided into three categories according to their roles in DNA methylation: writing enzymes, erasing enzymes, and reading enzymes. Writing enzymes catalyze the addition of methyl groups to cytosine residues. The function of erasing enzymes is to modify and remove methyl groups. Reading enzymes can recognize and bind methyl groups to affect gene expression. This figure was created with the aid of Biorender (https://biorender.com/). DNMTs DNA methyltransferases, MBD methyl-CPG-binding domain, ZBTB zinc finger and broad complex, tramtrack, and bric a brac, TET Ten Eleven Translocation, MECP2 methyl-CpG-binding protein 2, UNG uracil–DNA glycosylase

DNA methylation is a normal and universal modification in eukaryotic cells. It is also the main epigenetic form of gene expression regulation in mammals. There are several ways of methylated modification, but most of them occur on cytosine phosphate guanine (CpG) islands in the gene promoter region. DNA methylation is an important epigenetic mechanism. It can transfer genetic information to offspring DNA through the regulation of DNA methyltransferases (DNMTs) (Fig. 1).

DNA methylases can be divided into three categories according to their roles in DNA methylation: writing enzymes, erasing enzymes, and reading enzymes. Writing enzymes catalyze the addition of methyl groups to cytosine residues, including DNMT1, DNMT3a, and DNBT3b. Although these enzymes have large N-terminal regulatory domain and c-terminal catalytic domain, there are differences in function and expression patterns.^12^ DNMT1 can not only accurately mimic the original methylation pattern before DNA replication, but also repair DNA methylation.^13^ DNMT3a and DNMT3b are also known as de novo DNMT, which can introduce methylation into naked DNA and establish a new methylation pattern for unmodified DNA. DNMT3a and DNMT3b targeting specific DNA sequences may be mediated by transcription factors. They regulate de novo synthetic DNA methylation. DNMTs can bind components of transcription factors or repressor complexes to target DNA methylation.^14^ In addition, transcription factor binding can help protect CpG sites from de novo methylation. The function of eraser enzyme is to modify and remove methyl groups. DNA demethylation can occur either actively or passively. Passive demethylation means that maintenance of DNMTs during mitosis fails to methylate the newly synthesized DNA strand. However, the molecular mechanism that catalyzes active DNA demethylation has not been elucidated. Reading enzymes can recognize and bind methyl groups to affect gene expression. Proteins in the reading enzymes structural domain (adaptors) are mainly involved in gene expression. The main function of these structural domains is to recruit factors related to DNA metabolism progress, including DNA replication, transcription, DNA recombination, and DNA damage repair. Three protein families recognize DNA methylation: the methyl-CPG-binding domain (MBD) protein, the UHRF protein, and the zinc finger protein. MBD protein contains a conserved methyl-CPG-binding domain (MBD), which has a high affinity for a single methylated CpG site.^15^ The MBD family includes MeCP2, the first methyl-binding protein to be identified, as well as MBD1, MBD2, MBD3, and MBD4.^16^ The UHRF family of proteins maintains DNA methylation by binding to DNMT1 and targeting semi-methylated DNA.^17^ The zinc finger protein family consists of Kaiso, ZBTB4, and ZBTB38. It inhibits transcription mainly through DNA methylation-dependent mode.^18^

### Histone modification

**Fig. 2:**
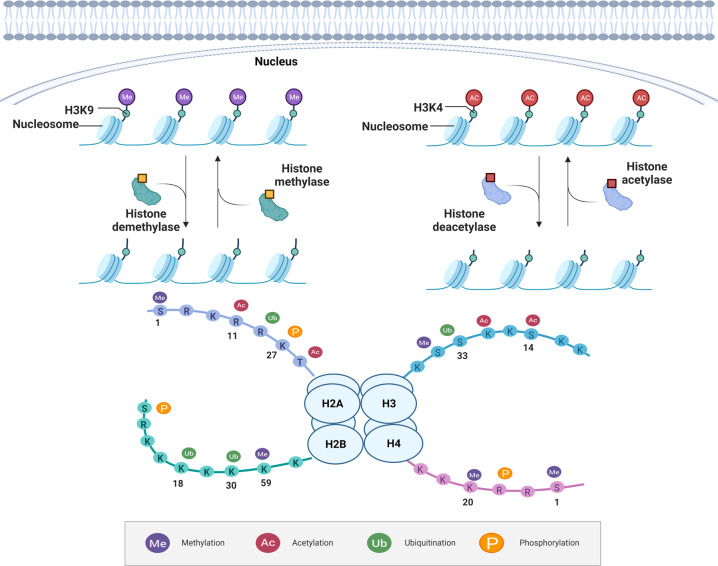
Histone-modification regulation. Histone modification refers to the process of histone modification such as methylation, acetylation, phosphorylation, adenylation, ubiquitination, and adenosine diphosphate ribosylation under the action of related enzymes. Among them, histone methylation and acetylation are well studied. Histone methyltransferases are mainly involved in the regulation of histone methylation, which transfers methyl groups to histones lysine residues, whereas, histone demethylases have the opposite effect. The methylation action site is on the N atom of the lysine side chain. Histone acetyltransferases are mainly involved in the regulation of histone acetylation, which transfers acetyl groups to histones lysine residues. However, histone deacetylases have the opposite effect. This figure was created with the aid of Biorender (https://biorender.com/). H3K9 histone H3 lysine 9, H3K4 histone H3 lysine 4

Histone modification refers to the process of histone modification such as methylation, acetylation, phosphorylation, adenylation, ubiquitination, and adenosine diphosphate ribosylation under the action of related enzymes. Modification of histones can change the loose or agglutinating state of chromatin by affecting the affinity between histones and DNA double strands. Gene regulation can also be performed by influencing the affinity between other transcription factors and structural genes promoters.^19^ Among them, histone methylation and acetylation are well studied (Fig. 2).

### Histone methylation

Histone methylation is one of the most important post-transcriptional modifications. Histone methyltransferase is mainly involved in the regulation of histone methylation, which transfers methyl groups to histones lysine residues using S-adenosine methionine as a substrate. The methylation site is on the N atom of the side chain of lysine and arginine. Common histone methylation includes H3K4 methylation, H3K9 methylation, and H3K27 methylation. Lysine methylation is relatively stable in gene expression regulation. The methylation of lysine residues at the fourth site of H3 is associated with gene activation, while the methylation of lysine residues at the ninth and 27th sites is associated with gene silencing. However, histone arginine methylation is a relatively dynamic marker. Arginine methylation is associated with gene activation, whereas loss of arginine methylation in H3 and H4 is associated with gene silencing. Histone H3K4 methylation mainly concentrates in regions of active transcription, such as the transcription start site, promoter, and enhancer regions. H3K4me1 was enriched in the enhancer region and correlated with H3K27ac or H3K27me3, marking active or inhibitory enhancers, respectively.^20^ Histone H3K9 methylation, especially H3K9me2 and trimethylated histone 3 lysine 9 (H3K9me3), generally regulates heterochromatin formation and gene inhibition.^21^ SUV39H1 and SUV39H2 catalyze the formation of H3K9me2 and H3K9me3.^22^ Enhancer of zeste homolog-2 (EZH2) can methylate H3K27, leading to silencing of related genes.

### Histone acetylation

Histone acetylation mainly occurs at the more conserved lysine sites at the N-terminus of H3 and H4. It is coordinated by histone acetyltransferases (HAT) and histone deacetylases (HDACs). Acetylation may regulate gene transcription through its effects on histone charges and interacting proteins. Therefore, histone acetylation is generally considered as an active histone marker. In 1996, researchers discovered the HAT P300 and cyclic AMP response element-binding protein (CBP).^23^ CBP binds to P300 to form the CBP/P300 complex, which recruits other HAT, such as PCAF (P300/CBP-related factor). According to the structural similarities and substrates of HDAC molecules, the HDACs family can be divided into four categories. HDAC1, 2, 3, and 8 belong to class I RPD3-like proteins. Class II HDACs can be divided into two subclasses: Class IIa (HDAC4, 5, 7, and 9) and Class IIb (HDAC6 and HDAC10). Class III HDACs are nicotinamide adenine dinucleotide (NAD+)-dependent deacetylases, mainly including Sirtuin (SIRT)1-SIRT7. Class IV protein is HDAC11. There are many lysine residues in histones that can be acetylated, such as H3K4, H3K9, H3K27, etc. Acetylation of lysine regulates functional changes in proteins by altering its structure or affinity for other binding partners. Therefore, lysine acetylation can regulate cancer,^24^ cardiovascular disease^25^ and other diseases. In the lysine acetyltransferase (KATs), P300 is a transcription coactivator that regulates gene expression by activating the intrinsic KAT.^26^ In addition, HDACs have been found to play an important role in the pathological processes of inflammation,^27^ cardiac hypertrophy, and heart failure.^28^

### Noncoding RNAs

**Fig. 3:**
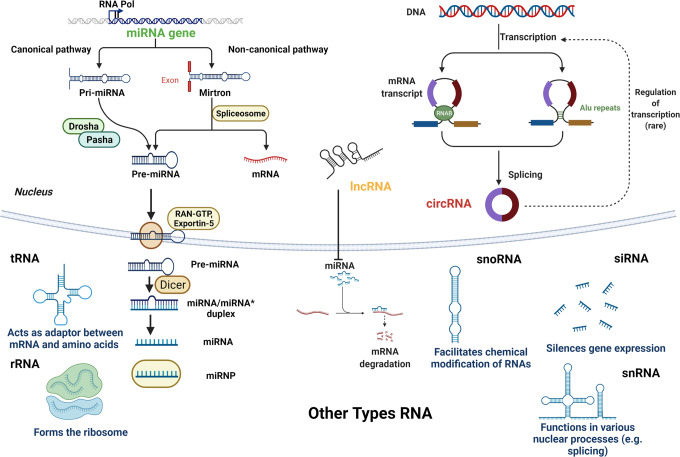
Noncoding RNA regulation. Different mechanisms of action of noncoding RNAs in epigenetic regulations. (1) <50 nt: MicroRNAs (miRNAs): miRNAs complement mRNAs and promote mRNA silencing or degradation. Small interfering RNAs(siRNA): silences gene expression. (2) 50–500 nt: nucleolar small RNA(snoRNA): snoRNA biological function was initially found to modify rRNA. Nuclear small RNA(snRNA): snRNA function is to combine with protein factors to form small nuclear ribonucleoprotein particle and perform the function of splicing mRNA. Transport RNA (tRNA): the main function is to carry amino acids into the ribosome and synthetic proteins with the guidance of mRNA. Ribosomal RNA (rRNA): it binds to proteins to form ribosomes.Its function is to act as a scaffold for mRNA, enabling mRNA molecules to unfold on it to achieve protein synthesis. (3) >500 nt: long noncoding RNAs (lncRNAs): LncRNA acts as mRNA and miRNA endogenous sponges regulating gene expression. Circular RNAs (cirRNAs): circRNA molecules are rich in miRNA-binding sites and act as miRNA sponges in cells, thereby lifting the inhibition of miRNA on target genes and increasing the expression level of target genes. This figure was created with the aid of Biorender (https://biorender.com/)

Noncoding RNAs refer to RNAs that do not encode proteins, including ribosomal RNAs, transport RNAs, small nuclear RNAs, small nucleolar RNAs, microRNAs (miRNAs), mRNA, and other known functions, as well as those with unknown functions. These RNAs have common feature that they can be transcribed from the genome but not translated into proteins, performing their respective biological functions at the RNA level (Fig. 3). Noncoding RNAs can be divided into three categories according to length: <50 nt: miRNA, small interfering RNAs, etc. 50–500 nt: ribosomal RNA, transport RNA, nuclear small RNA, nucleolar small RNA, etc. >500 nt: long mRNA-like ncRNAs, long noncoding RNAs (lncRNAs) without polyadenylate tails, etc.

#### miRNAs

miRNAs are a major class of small ncRNAs, about 22 nucleotides in length, found in animals, plants, and some viruses. Most miRNAs genes are transcribed primarily by RNA polymerase II (Pol II) into large primary miRNAs (PRI‐miRNAs), which contain one or several stem ring structures, each consisting of about 70 nucleotides. MiRNAs play a role in the post-transcriptional regulation of gene expression. Transcription levels are regulated by tissue-specific epigenetic modifications.^29^ Thus, miRNA genes are not only targets of epigenetic modifications such as DNA methylation, but also regulators of DNMTs and HDACs.^29^ miRNAs not only occupy a very important position in the progression of cardiovascular disease, such as cardiac hypertrophy and myocardial cell fibrosis,^30^ miRNAs can also serve as therapeutic targets for disease.^31^

#### LncRNAs

LncRNAs are nucleotides over 200 in length that cannot be converted into proteins. LncRNAs based on function mechanism that are divided into activated ncRNAs with enhancer-like properties (ncRNA-a), competitive endogenous RNAs (ceRNAs), primary transcripts for mi- and piRNAs. NcRNAs-a positively regulate nearby genes, which are the main difference from enhancer RNAs (eRNAs). CeRNAs share a sequence with the transcript encoding the protein. CeRNAs can competitively bind its regulated molecules to perform better function.^32^ LncRNA regulates gene expression patterns by altering chromatin structure and DNA accessibility through molecular mechanisms such as signaling, bait, guidance and scaffolding.^33^ LncRNAs have high functional specificity by participating in and regulating various cellular processes such as DNA methylation and histone modification.^34^ Furthermore, LncRNAs not only regulate cardiovascular diseases, but also are increasingly attached importance in the progression of blood-related diseases.

#### Circular RNAs

Circular RNAs (CirRNAs) are long, noncoding endogenous RNA molecules with single-stranded covalently closed RNA loops without 5'-Cap and 3’-poly(A) ends. With the development of sequencing technology, several types of cirRNAs have been discovered and identified. There are four main subtypes: exon cirRNAs (ecircRNAs), which are mainly derived from single or multiple exons; circular intron-type cirRNAs, which contain only introns; exon–intron-type cirRNAs (EIciRNAs), which consist of exons and introns. At present, most cirRNAs identified are ecircRNAs.^35^ Accumulated data suggest that cirRNAs exert their regulatory functions through the following mechanisms: (1) acting as miRNA sponges. CirRNAs regulate the expression of target genes and mRNA translation by interacting with miRNA. (2) as a protein scaffold. CirRNAs can bind to RNA binding proteins or functional proteins to regulate their function and transport. (3) as an important molecule of transcriptional regulation. CircEIF3J and circPAIP2 interact with U1snRNA and RNA polymerase II complexes to enhance transcriptional activity. (4) as a template for protein synthesis. It can participate in protein translation.^36^ CirRNAs are widespread and diverse in eukaryotic cells,^37^ but relatively stable in the cytoplasm.^38^ CirRNAs can be co-transcribed and post-transcribed by a process of reverse splicing or head-tail circular splicing, in which the downstream exon splices in reverse order to the upstream exon.^39^ CirRNAs are transformed into linear RNA by targeting miRNA and regulate gene expression. CirRNA is more stable than linear RNA isoforms because it lacks an accessible terminal that can resist RNA exonuclease. The functional mechanism of cirRNA is thought to be to change the level of free miRNA in sponges by interacting with miRNA, and then regulate the expression of disease-related proteins.^40^ In addition, cirRNA is involved in regulating the progression of cardiovascular and autoimmune diseases.^40–42^ Therefore, cirRNA can be used as one of the potential strategies for clinical diagnosis and disease treatment. The epigenetic regulatory mechanisms have been summarized above (Table 1). In the third part, we mainly discuss the research progress of epigenetics in cardiovascular disease in recent years.

**Table 1:** A brief summary of epigenetics

Epigenetics	Definition	Classification	Key enzyme	Major function
DNA methylation	Under the catalysis of DNA methyltransferase, the methyl of S-adenosine methionine is transferred to DNA sequence	5-hydroxymethyl cytosine (5hmC), 5-methyl cytosine (5mC), N6-methyl adenine (6 mA) and 7-methyl guanine (7mG) and other forms	DNMT1, DNMT3a/b	DNA methylation level has an important influence on gene expression. In general, the promoter region of active genes is in the state of demethylation, while the promoter region of silenced genes is in the state of hypermethylation.
Histone modification	It refers to the modification process in which histones regulate methylation, acetylation, phosphorylation, adenylation, ubiquitination and ADP ribosylation through the action of related enzymes.	Histone methylation, acetylation, phosphorylation, ubiquitination, deamidation, ADP ribosylation and proline isomerization	Histone methylase, histone demethylase, Histone acetylase, histone deacetylase, Histone phosphorylase, Histone ubiquitination enzyme	Different histone modifications regulate gene expression and guide cell differentiation by activating or inhibiting transcription.
Noncoding RNA	It refers to functional RNA molecules in the transcriptome that do not encode proteins	microRNAs, small interfering RNAs, ribosomal RNA, transport RNA, nuclear small RNA, nucleolar small RNA, long mRNA-like ncRNAs, long noncoding RNAs without polyadenylate tails, circular RNA	–	miRNA regulate specific genes expression by inducing degradation of target mRNA or interfering with protein translation process. LncRNA regulates gene expression patterns by altering chromatin structure and DNA accessibility through molecular mechanisms. CirRNAs: (1) acting as miRNA sponges. (2) as a protein scaffold. (3) as an important molecule of transcriptional. regulation. (4) as a template for protein synthesis.

### Epigenetic regulatory mechanisms in cardiovascular disease

**Fig. 4:**
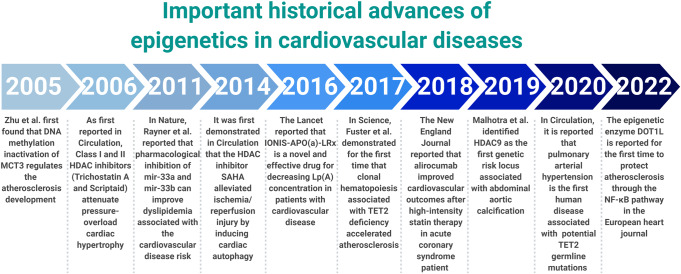
Important historical advances of epigenetics in cardiovascular diseases. This figure was created with the aid of Biorender (https://biorender.com/). MCT3 monocarboxylate transporter 3, HDAC histone deacetylase, SAHA suberoylanilide hydroxamic acid, Lp(A) lipoprotein(A), TET2 TET-methylcytidine dioxygenase-2, DOT1L disruptor of telomeric silencing 1-like, NF-kB nuclear transcription factor-kappa B

In recent years, epigenetics has been occupied an indispensable position in the cardiovascular diseases historical development progress. The correlation of epigenetics with cardiovascular diseases has primarily been identified in the function and expression of epigenetic-related enzymes found in cardiovascular diseases. To better understand the discovery and research history of epigenetics in cardiovascular diseases, it is helpful to review the timeline of epigenetics^43–63^ (Fig. 4).

### The role of DNA methylation in cardiovascular disease

Several studies have shown that DNA methylation plays important roles in cardiovascular diseases (Table 2). In recent years, it has been found that the expression of candidate genes related to coronary heart disease, heart failure, hypertension, and other cardiovascular diseases is associated with DNA methylation. The abnormal methylation status of candidate genes is involved in the mechanism and development of cardiovascular disease and can be used as a marker to assess cardiovascular disease progression.

**Table 2:** Regulation of DNA methylation in cardiovascular disease

Diseases	Target gene	Detection	Effect	References
Coronary heart disease	cg22304262	DNA-specific locus	–	^65^
Coronary heart disease	Whole Genome	Global DNA	–	^67^
Coronary heart disease	Whole Genome	Global DNA	–	^69^
Coronary heart disease	cg04988978, MPO	DNA-specific locus	Promote	^70^
Acute myocardial infarction	Whole Genome	Global DNA		^68^
Heart failure	DNMT3a	DNA-specific locus	Inhibit	^75^
Heart failure	CTGF, MMP2, miRNA-155, HEY2, MSR1, MYOM3, COX17, miRNA-24-1	DNA-specific locus	–	^76^
Heart failure	KCNA4, KCNIP4, SMOC2	DNA-specific locus	Promote	^77^
Heart failure	DNMT2, glutathione peroxidase 1	DNA-specific locus	Inhibit	^78^
Vascular calcification	DNMT3b, H19	DNA-specific locus	Promote	^79^
Vascular calcification	G3BP1	DNA-specific locus	Promote	^80^
Vascular calcification	SM22a	DNA-specific locus	Promote	^81^
Vascular calcification	miR-34b	DNA-specific locus	Inhibit	^82^
Vascular calcification	miR-204	DNA-specific locus	Inhibit	^83^
Hypertension	mitochondrial fusion 2	DNA-specific locus	Promote	^86^
Hypertension	interferon γ	DNA-specific locus	Promote	^87^
Tetralogy of Fallot	cg05273049, cg02540011, cg08404201 cg00687252	DNA-specific locus	–	^212^

*MPO* myeloperoxidase, *CTGF* connective tissue growth factor, *MMP* matrix metalloproteinase, *HEY2* hairy and enhancer-of-split related with YRPW motif 2, *COX17* cytochrome c oxidase subunit 17, *BMP2* bone morphogenetic protein 2, *G3BP1* GTPase-activating protein-binding protein, *SM22a* smooth muscle 22 alpha

#### Coronary heart disease and myocardial infarction

In genome-wide Bonferroni multiple assay correction, Westerman et al.^64^ found that DNA methylation in three regions (associated with SLC9A1, SLC1A5, and TNRC6C genes) was associated with cardiovascular disease risk. Mendelian randomization analysis showed that one CpG (CG22304262) in SLC1A5 had a causal relationship with incident coronary heart disease (iCHD). The DNA methylation level of CG22304262 may affect SLC1A5 expression.^65^ Myocardial glutamine storage disorder and SLC1A5 expression were decreased in patients with heart failure. Inhibition of SLC1A5 expression in the myocardium reduced glutamine uptake and impaired glutamine homeostasis in failing myocardium.^66^ In conclusion, this study explores new blood DNA methylation regions associated with iCHD and has important implications for improving clinical risk prediction.^64^ In iCHD’s latest Epigenome-wide association studies, Navas-Acien et al.^67^ identified a biological association between differentially methylated position and atherosclerosis. In this study, blood DNA methylation was associated with coronary heart disease over and above the traditional factors associated with cardiovascular disease. Meanwhile, there were large differences and complex epigenomic characteristics in different populations.^67^

A study performed a comprehensive analysis of DNA methylation and mRNA expression datasets at a series of time points in a mouse model of acute myocardial infarction (AMI). It was found that the most critical stage of AMI was 6 h. A large number of methylation modification sites were changed during this stage. Five genes (Ptpn6, Csf1r, Col6a1, Cyba, and Map3k14) were identified to participate in AMI process by regulating DNA methylation. These candidate genes are expected to be methylated biomarkers for early clinical diagnosis of acute myocardial infarction in future.^68^ Framingham offspring’s cohort study of DNA methylation found that four independent underlying factors (9, 19, 21, 27) driven by DNA methylation were associated with cardiovascular disease. In addition, three genes contained in factor 27 were also associated with myocardial infarction factors (CDC42BPB, MAN2A2, and RPTOR). Recent multifactorial approaches integrating DNA methylation and gene expression data provide new insights into the pathogenesis of the cardiovascular disease.^69^ Thirty-four new DNA methylation sites associated with AMI were identified in two-stage Epigenome-wide association studies. Four of them were associated with coronary heart disease. Cg21566642 was located in the intergenic region, cg05575921, cg04988978, and cg25769469 were labeled AHRR, MPO, and PTCD2, respectively. MPO encodes myeloperoxidase, which promotes atherosclerotic lesions by enhancing APOB oxidation in low-density lipoprotein (LDL)^70^ and is causally associated with cardiovascular events.^71^ A CpG located in PTCD2 has previously been associated with hypertension in patients with obstructive sleep apnea.^72^ Genetic variants of this gene have been associated with blood pressure.^73^ These differential methylated genes are enriched in various molecular and physiological pathways such as lipid metabolism and inflammatory diseases. They are closely related to the pathogenesis of coronary heart disease and AMI.^74^

#### Heart failure

Recently, the regulatory role of DNA methylation in cardiac hypertrophy and heart failure has attracted much attention. However, its exact role in cardiomyocytes remains controversial. Madsen et al.^75^ showed that DNA methylation of DNA methyltransferase DNMT3a was involved in the homeostasis of human cardiomyocytes. Knockout of DNMT3a not only changed the expression of contractile protein gene in cardiomyocytes, but also resulted in cardiomyocytes mitochondrial damage and impaired glucose metabolism. Therefore, regulating the abnormal DNA methylation process is of great significance for further understanding heart failure pathogenesis.^75^ Glezeva et al.^76^ detected 195 distinct regions of differential methylation in a cohort studying heart failure, primarily distributed in hypertrophic obstructive cardiomyopathy, dilated cardiomyopathy, and ischemic cardiomyopathy. In addition, five genes (HEY2, MSR1, MYOM3, COX17, and miRNA-24-1) were found to be hypermethylated in the ventricular septal tissues of heart failure patients that included hypertrophic obstructive cardiomyopathy, ischemic cardiomyopathy, and dilated cardiomyopathy. Three genes (CTGF, MMP2, and miRNA-155) showed hypomethylated state. This study supports the role of DNA methylation in the regulation of heart failure-related genes for different clinical causes. Therefore, these differentially expressed methylated genes in heart failure may be a new markers for the detection and diagnosis of heart failure.^76^ A study on the effect of genome-wide cardiac DNA methylation on overall gene expression in myocardial samples from patients with end-stage chronic Chagas disease cardiomyopathy (CCC) showed that two differentially expressed methylated genes, KCNA4 and KCNIP4, were involved in the regulation of potassium channels. They were upregulated in CCC and encoded potassium voltage-gated channels Kv1.4 and Kv4.3 to participate in electrical conduction and arrhythmias. The protein encoded by the differentially expressed methylated gene SMOC2 was upregulated in CCC and was involved in CCC matrix remodeling and fibrosis. Therefore, DNA methylation can reveal the pathogenesis and process of CCC by regulating CCC-related cardiac and immune system genes expression.^77^ In addition, Zhu et al.^78^ demonstrated that selenium supplementation could inhibit DNMT2-induced DNA methylation of glutathione peroxidase 1 gene promoter in cardiomyocytes, increased glutathione peroxidase 1 expression, further reduced intracellular reactive oxygen species production, and cardiomyocyte apoptosis, and thus played a protective role in heart failure.^78^

#### Vascular calcification

DNA methylations related molecules are considered to have the potential to be used as biomarkers for the diagnosis of vascular calcification. Dai et al.^79^ demonstrated that the expression and activity of S-adenosylhomocysteine hydrolase (SAHH) were reduced in calcified human coronary arteries. SAHH deficiency increased intracellular S-adenosylhomocysteine (SAH) levels, leading to hypomethylation and upregulated expression of H19 gene promoter through competitive inhibition of DNMT3b, Thus promoting H19-mediated runt-related transcription factor-2 (Runx2)-dependent vascular smooth muscle cell (VSMC) osteogenic differentiation and aggravating atherosclerotic calcification. In contrast, SAHH lacks reduced intracellular adenosine levels and AMPK (AmP-Activated protein kinase) activation. Adenosine supplementation activated AMPK. AMPK eliminated H19-mediated Runx2-dependent VSMC osteogenic differentiation by inducing sirt1-mediated low acetylation of histone H3 and DNMT3b-mediated hypermethylation of H19 promoter.^79^ Ramachandran et al.^80^ demonstrated that GTPase-activating protein-binding protein (G3BP1) n-terminal nuclear transporter-2 and c-terminal arginine methylation domains were important in activating osteogenic-related transcriptional reactions. G3BP1 methylation was enhanced by the knockout of the typical Wnt receptor LRP6 in mouse aortic vascular smooth muscle (VSM). It was accompanied by activation of osteogenic transcription programs mediated in part by Runx2. Furthermore, G3BP1 activated the transcription of activated T cells nuclear factor (namely NFATc4), and then promoted VSM NFATc4 association with osteopontin (OPN) and alkaline phosphatase (ALP) promoters. Thus G3BP1 could accelerate calcification process.^80^ High phosphate increases DNMTs activity in the smooth muscle 22a (SM22a) promoter region. SM22a promoter methylation reduces the SM22a gene expression, promotes the osteoblast transcription factor core-binding factor 1 expression and the ALP activity upregulation. Finally, SM22a promoter methylation leads to the VSMCs to osteoblast phenotype transformation and calcification.^81^ In addition, a study showed that miRNA-34b expression was significantly inhibited in VSMCs treated with high inorganic phosphate. Inhibition of miRNA-34b expression can enhance VSMCs calcification. Elevated DNMT3a induced miRNA-34b hypermethylation in VSMCs and reduced miRNA-34b expression, thereby promoting the occurrence of calcification. After DNMT3a siRNA knockout, the effect of high phosphate on VSMCs calcification disappeared. This is because DNMT3a knockout reduces the miRNA-34b methylation, and the expression level of miRNA-34b increases, acting on its downstream target Notch 1 and reducing VSMCs calcification.^82^ Lin et al.^83^ reported that in high phosphate-induced VSMCs, the increase of DNMT3a leaded to miRNA-204 hypermethylation and expression downregulation, thereby promoting VSMCs osteogenic differentiation. After DNMT3a small interfering RNA knockout DNMT3a, miRNA-204 expression was upregulated and VSMCs osteogenic differentiation was alleviated. Thus it eliminates the effect of high phosphate treatment on VSMC calcification.^83^

#### Hypertension

DNA methylation was shown to have an important function in hypertension development. A study exploring the association of whole blood DNA methylation with 24-h blood pressure phenotype and clinical blood pressure reported that 72 DNA methylation regions (MRs) were identified as significantly associated with 24-h blood pressure phenotypes (24-h mean, day and night) out of 1,549,368 CpG sites.^84^ Dwi Putra et al.^85^ found that mothers with a family history of hypertension had higher mean arterial pressure and lower overall placental DNA methylation in placental samples compared with mothers without a family history of hypertension. However, only in mothers with no family history of hypertension, overall placental DNA methylation was independently negatively associated with maternal mean arterial pressure.^85^ Jin et al.^86^ found that mitochondrial fusion 2 can inhibit VSMCs proliferation and is closely related to inflammation, oxidative stress and renin angiotensin system (RAS). Correlation analysis showed that mitochondrial fusion 2 gene methylation level was significantly lower in hypertensive patients than in the control group. Mitochondrial fusion 2 hypomethylation may downregulate the expression of this gene. Consequently, it led to VSMCs proliferation and endothelial cell damage, and then contributed to the development of hypertension.^86^ Bao et al.^87^ study result showed that hypomethylation of the interferon γ gene can induce vascular endothelial chemotaxis in a long-term inflammatory state hypertensive patients. At the same time, in vascular tissue, hypomethylation of the interferon γ gene increased VSMCs proliferation and lipid deposition. Therefore, it gave rise to the transformation of blood vessels from normal phenotype to vascular fibrosis, resulting in increased blood pressure.^87^ In conclusion, these studies indicate that DNA methylation is closely related to the occurrence of cardiovascular diseases.

### The role of histone modification in cardiovascular disease

Histone modification is one of the important regulatory mechanisms in epigenetics. Abnormal histone modification results in an imbalance in the expression of genes associated with cardiovascular disease, resulting in changes in cellular phenotypes and cardiac function. Key molecules of histone modification (histone methylation and histone acetylation) may lead to the occurrence and progression of cardiovascular disease through their influence on cardiovascular pathophysiological pathways (Table 3).

**Table 3:** Regulation of histone modification in cardiovascular disease

Diseases	Types of histone modifications	Major regulator	Target gene	Effect	References
Myocardial hypertrophy	Histone methylation	Histone methyltransferase G9a	Histone 3 lysine 9 EZH2	Inhibit	^88^
Vascular calcification	Histone methylation	IL-6/SIL-6R	Histone 3 lysine 9, JMJD2B	Promote	^89^
Vascular calcification	Histone methylation	EZH2	TAGLN	Promote	^90^
Atherosclerosis	Histone acetylation	SIRT1	eNOS	Inhibit	^91–93^
Atherosclerosis	Histone acetylation	HDAC3	eNOS	Promote	^91–93^
Atherosclerosis	Histone acetylation	SIRT1	P65, P300, NF-κB	Inhibit	^96^
Myocardial infarction	Histone acetylation	SIRT2	FOXO3A	Promote	^99^
Myocardial infarction	Histone acetylation	SIRT3	Cyclophilin D	Inhibit	^100^
Myocardial infarction	Histone acetylation	HDAC6	Peroxyredoxin 1	Promote	^101^
Heart failure	Histone acetylation	SIRT2	Angiotensin II	Inhibit	^102^
Heart failure	Histone acetylation	SIRT3	GSK3β, SMAD3	Inhibit	^103^
Heart failure	Histone acetylation	SIRT4	angiotensin II	Promote	^104^
Heart failure	Histone acetylation	SIRT6	P300	Inhibit	^105^
Heart failure	Histone acetylation	SIRT1	NOTCH1	Promote	^108^
Heart failure	Histone acetylation	SIRT7	p53	Inhibit	^110^
Vascular calcification	Histone acetylation	SIRT6	Runx2	Inhibit	^111^
Vascular calcification	Histone acetylation	HDAC4	Sox9, Runx2, ALP	Promote	^112^
Vascular calcification	Histone acetylation	HDAC9	Runx2	Promote	^59^
Vascular calcification	Histone acetylation	SIRT1	RUNX2, osteocalcin	Inhibit	^113^
Hypertension	Histone acetylation	HDAC6	CSEγ	Promote	^114^
Hypertension	Histone acetylation	SIRT3	SOD2	Inhibit	^116^
Hypertension	Histone acetylation	HDAC1/2	Npr1	Inhibit	^117^
Atrial fibrillation	Histone acetylation	HDAC2	KCNJ2 K + ion channel	Promote	^213^

*EZH2* enhancer of zeste homolog-2, *IL-6* interleukin 6, *SIRT1* Sirtuin1, *eNOS* endothelial nitric oxide synthase, *HDAC* histone deacetylase, *FOXO3A* forkhead box O3A, *Runx2* runt-related transcription factor 2, *ALP* alkaline phosphatase, *SOD2* superoxide dismutase 2, *CSEγ* cystathionine γ-lyase, *Npr1* encoding natriuretic peptide receptor-A

### The role of histone methylation in cardiovascular disease

Cardiovascular disease can also be regulated by histone methylation (Table 3). Papait et al.^88^ found that histone methyltransferase G9a had a synergistic effect with the catalytic subunit EZH2 of PRC2 on gene silencing. G9a inhibited cardiomyocytes' different types gene function through dimethylation of lysine 9 on histone H3 and interaction with EZH2. Therefore, G9a was essential to maintain correct gene expression in normal cardiomyocytes and to drive changes in the expression of genes associated with cardiac hypertrophy. The study results suggest that impaired G9a function can lead to cardiac dysfunction. G9a may be a potential target for the treatment of early myocardial hypertrophy in the future.^88^ Kurozumi et al.^89^ showed that interleukin (IL)-6/SIL-6R stimulation induces p-STAT3 activation and jumonji domain-containing protein (JMJD)2B recruitment. Runx2 gene expression is increased in human VSMCs (hVSMCs) by bivalent histone modification of the transcription enhancer trimethylation of lysine 4 of histone H3 (H3K4me3) and the transcription suppressor trimethylated histone 3 lysine 9 (H3K9me3). JMJD2B protein was highly expressed in hVSMCs. IL-6/SIL-6R stimulation may result in the recruitment of JMJD2B to stat-targeted sites in the Runx2 promoter region, which demethylated H3K9me3. Therefore it increased the osteoblast differentiation markers (ALP and OPN mRNA) expression, and then enhanced osteoblast differentiation and vascular calcification.^89^ SM22a encoded by Transgelin (TAGLN) is expressed in mesenchymal cells such as smooth muscle cells. Maleszewska et al.^90^ reported that TAGLN/SM22a expression was regulated at the epigenetic level by polycomb methyltransferase EZH2. Downregulated IL-1β and transforming growth factor-β (TGFβ)-2 increased the EZH2 expression, inhibited the TAGLN expression, and increased the H3K27me3 level at the proximal promoter of TAGLN. EZH2 regulated the chromatin structure of the TAGLN promoter through trimethylation of H3K27. In addition, activation of EZH2 decreased TGFβ2-induced SM22a and TAGLN expression. SM22a is essential for the maintenance of smooth muscle cell phenotype and function, which may lead to calcification.^90^ In conclusion, these studies suggest that histone methylation is strongly associated with cardiovascular disease physiopathologic mechanism.

### The role of histone acetylation in cardiovascular disease

HATs and HDACs play a crucial effect in regulating histone acetylation. The role of HAT and HDACs-mediated epigenetic processes in vascular homeostasis and cardiovascular disease has received extensive attention (Table 3).

#### Atherosclerosis and myocardial infarction

Research has found that SIRT1 inhibited the formation of atherosclerotic plaques in ApoE−/− mice by regulating endothelial nitric oxide synthase (eNOS) activation. Thus SIRT1 slowed down the formation of atherosclerosis. However, HDAC3-mediated deacetylation of eNOS K610 promoted atherosclerosis.^91–93^ The level of inducible nitric oxide synthase (iNOS) is also increased in atherosclerotic lesions.^94^ In endothelial cells, nuclear factor-κB (NF-κB) promotes atherosclerosis by driving the expression of Nos2(encoding iNOS) and proinflammatory genes.^95^ SIRT1 inhibits NF-κB activity by deacetylating P65 and eliminating the interaction between P300 and NF-κB.^96^ It has been reported that lysine acetylation can regulate myocardium ischemia–reperfusion injury and myocardial infarction. Overexpression of SIRT1 can alleviate ischemia–reperfusion injury in rat myocardium.^97,98^ SIRT2 promotes cell apoptosis in renal ischemia–reperfusion injury by deacetylating Forkhead box O3A (FOXO3A).^99^ SIRT3 can inhibit reperfusion injury by deacetylation of cyclophilin D and prevent the opening of mitochondrial permeability transition pores, resulting in cell death.^100^ In addition, in rat models of myocardial ischemia–reperfusion injury, HDAC6 reduces the activity of peroxyredoxin 1 by deacetylating its K197 site. In the end, it leads to increased reactive oxygen species production and exacerbate oxidative damage of cardiomyocytes.^101^

#### Heart failure

Heart failure is characterized by dysapoptosis of myocardial cells, increased fibrotic scar tissue, and pathological myocardial hypertrophy. SIRT2 deficiency exacerbates angiotensin II-induced myocardial fibrosis.^102^ SIRT3 can activate GSK3β through K15 deacetylation, which in turn phosphorylates SMAD3 and leads to its degradation, thereby preventing TGF β-induced fibrosis.^103^ In contrast, SIRT4 depletion attenuated angiotensin II-induced myocardial fibrosis.^104^ Shen et al.^105^ found that SIRT6 levels were significantly reduced in phenylephrine-induced neonatal rat cardiomyocytes, which was associated with increased acetyltransferase P300 levels and cardiomyocyte hypertrophy. Overexpressed SIRT6 attenuated phenylephrine-induced cardiac hypertrophy by degrading P300.^105^ In vitro, knockdown of HDAC4 in human lung fibroblasts inhibits myofibroblast differentiation. Nevertheless, the knockdown of HDAC6 in rat heart fibroblasts blocks cell proliferation.^106^ These findings suggest that histone acetylation regulates myocardial cell fibrosis in the course of heart failure.

Pathological cardiac remodeling in heart failure is associated with dysregulated myocardial apoptosis.^107^ SIRT1 overexpression increased apoptosis of neonatal rat cardiomyocytes in vitro by reversing the acetylation of the isodimer receptor NOTCH1. Thus, the stability of NOTCH1 was decreased and the proliferation of cardiomyocytes was inhibited.^108^ However, overexpression of SIRT2 significantly increased cardiomyocyte hypertrophy, but protected cardiomyocytes from apoptosis under serum starvation in vitro.^109^ SIRT7 inhibited myocardial apoptosis in vitro by deacetylating p53 and increasing stress resistance.^110^ These results suggest that histone acetylation may be involved in the proliferation and apoptosis of cardiomyocytes.

#### Vascular calcification

In recent years, increasing evidence has accumulated for histone acetylation in vascular calcification progress. Li et al.^111^ showed that SIRT6 can deacetylate Runx2, further promoted Runx2 to go out of the nucleus in an exportin 1-dependent manner, and then degraded Runx2 through the ubiquitin–proteasome system, thereby reducing osteogenic differentiation of VSMCs and inhibiting vascular calcification. Therefore, this study illustrates the new potential of longevity protein SIRT6 in the treatment of vascular calcification, which provides a new intervention target and strategy for its clinical prevention and treatment.^111^ Abend et al.^112^ found that HDAC4 was upregulated in the early stage of VSMCs calcification. HDAC4 binding and its activity-induced osteocalcin upregulation in vitro VSMCs and aortic rings via the adaptor protein ENIGMA (Pdlim7). Overexpression of HDAC4 can upregulate SOX9, Runx2, ALP, proteoglycan, and calcium mineral accumulation. Therefore, these data identify HDAC4 as a positive regulator of the vascular calcification process.^112^ HDAC9, a member of the HDAC IIa family, also plays a role in vascular calcification. Malhotra et al.^59^ reported that HDAC9 increased expression in human aortic smooth muscle cells and promoted osteogenic phenotype and calcification of VSMCs by upregulating Runx2 gene expression. These results suggest that HDAC9 may be a potential therapeutic target for vascular calcification in the future. However, the exact mechanism by which HDAC9 regulates vascular calcification needs further study.^59^ In addition, Bartoli-leonard et al.^113^ reported that SIRT1 activation inhibited smooth muscle cells osteogenic transdifferentiation and reduced the diabetic vascular calcification progression by downregulating the Runx2 and osteocalcin expression.^113^

#### Hypertension

Existing studies have shown that histone acetylation is closely related to the occurrence and development of hypertension. A study to investigate the role of HDAC6 in hypertension found that Ang II upregulated HDAC6 mRNA and protein expression. HDAC6 induced the deacetylation of Cystathionine γ-lyase (CSEγ), leading to CSEγ degradation through the ubiquitin–albumin pathway. CSEγ is the main enzyme in the production of physiological vasodilator hydrogen sulfide. The degradation of CSEγ reduced the production of hydrogen sulfide, which accelerated hypertension and impaired endothelial function.^114^ Several studies have shown that changes in HDACs, SIRT1, SIRT3, and bromodomain-containing protein 4 (BRD4) protein expression levels are associated with cell proliferation, inflammation, and pathological vascular remodeling, thereby regulating the pathological processes of hypertension and pulmonary hypertension.^115^ Downregulation of SIRT3 expression and SIRT3 redox inactivation result in superoxide dismutase 2 (SOD2) inactivation, which promotes the occurrence of hypertension.^116^ In addition, Arise et al.^117^ found that angiotensin II(Ang II) enhanced the activity of class I HDAC1/2, reduced histone acetylation of H3K9/14ac and H4K8ac, further inhibited Npr1 (encoding natriuretic peptide receptor-A) transcription, and decreased natriuretic peptide receptor-A protein and cGMP levels, thereby diminishing renal and vascular reactivity and attenuating atrial natriuretic peptide-mediated aortic ring relaxation. Therefore, the study suggests that Ang-II-mediated Npr1 transcription and receptor function inhibition may provide new molecular targets and an important theoretical basis for the treatment and prevention of hypertension.^117^ In conclusion, these studies indicate that histone acetylation is closely related to cardiovascular diseases mechanism.

### The role of noncoding RNAs regulation in cardiovascular disease

A large number of studies have shown that noncoding RNAs plays a key regulatory role in cardiovascular disease. Identification of specific noncoding RNAs will provide new ideas and directions for early diagnosis and prevention of diseases. So far, many noncoding RNAs have been found to be associated with the physiological and pathophysiological processes of cardiovascular diseases such as coronary heart disease, myocardial infarction, and vascular calcification. Noncoding RNA expression is cell- and organ-specific. Noncoding RNAs related to cardiovascular diseases exist in human blood, urine, and other body fluids. Moreover, due to its high sensitivity, stability, easy acquisition, and detection, it is expected to be a novel biomarker for assessing the risk stratification, diagnosis, and prognosis of cardiovascular disease in the future.

### The role of miRNAs in cardiovascular disease

In recent years, a number of studies have confirmed that miRNAs can regulate the pathophysiological process of cardiovascular diseases (Table 4).

**Table 4:** Regulation of noncoding RNA in cardiovascular disease

Diseases	Types of noncoding RNAs	Major regulator	Target gene	Effect	References
Coronary heart disease	miRNA	miRNA-SNP rs41291957	miRNA-143, miRNA-145	Inhibit	^118^
Atherosclerosis	miRNA	miRNA-1	KLF4	Promote	^119^
Atherosclerosis	miRNA	miR-92a	KLF4	Promote	^122^
Acute myocardial infarction	miRNA	miR-125b	SIRT7	Inhibit	^123^
Acute myocardial infarction	miRNA	miR-21a-5p	PDCD4, PTEN, Peli1, FasL	Inhibit	^124^
Acute myocardial infarction	miRNA	miR-25-3p	E2Z2	Inhibit	^125^
Acute myocardial infarction	miRNA	miR-144	PTEN/AKT	Inhibit	^126^
Heart failure	miRNA	miR-425, miR-744	TGF-β	Inhibit	^132^
Vascular calcification	miRNA	miRNA-30b	MMPs, SOX9	Inhibit	^133,134^
Vascular calcification	miRNA	miRNA-128‐3p	Wnt‐1, β‐catenin, GSK‐3β, Bax, Islet1	Promote	^136^
Vascular calcification	miRNA	miRNA-19A-3p	HDAC4	Promote	^137^
Pulmonary arterial hypertension	miRNA	miR-181a-5p, miR-324-5p	Norch4, ETS1	Inhibit	^138^
Hypertension	miRNA	miR-181a-5p, miR-663	renin	Inhibit	^140^
Metabolic cardiomyopathy	miRNA	miRNA-494-3p	JunD/PPARγ	Inhibit	^215^
Cardiomyocyte differentiation	LncRNA	Linc1405	Eomes, MesP1	Promote	^141^
Cardiac regeneration and differentiation	LncRNA	LncRNA CAREL	MiR-296	Inhibit	^145^
Cardiac regeneration	LncRNA	LncRNA NR_045363, Sirt1 antisense LncRNA	miRNA-216a, Sirt1 mRNA	Promote	^146,147^
Atherosclerosis	LncRNA	LncRNA Mexis	ABCA1	Inhibit	^159^
Atherosclerosis	LncRNA	LncRNA NEXN-AS1	TLR-4 oligomer, NF-κB	Inhibit	^160^
Myocardial infarction	LncRNA	lncRNA Gpr19	mir-324-5p, Mtfr1	Promote	^166^
Myocardial infarction	LncRNA	lncRNA UCA1	Mir-143, MDM2, p53	Inhibit	^167^
Heart failure	LncRNA	lncRNA Meg3	MMP2	Promote	^175^
Heart failure	LncRNA	lncRNA Whisper	Col3a1, Fn1, Tgfb2, aSma	Promote	^176^
Vascular calcification	LncRNA	LncRNATUG1	miRNA-204-5p	Promote	^177^
Vascular calcification	LncRNA	Lrrc75a-as1	SRF, CREB1, STAT3	Inhibit	^178^
Vascular calcification	LncRNA	LncRNA-SNHG29	miR-200b-3p	Inhibit	^179^
Vascular calcification	LncRNA	Bhlhe40 lncRNA-ES3	miR-95-5p, miR-6776-5p, miR-3620-5p, miR-4747-5p	Inhibit	^180^
Hypertension	LncRNA	HAS2-AS1	C/EBPβ	Promote	^181^
Hypertension	LncRNA	MRAK048635_P1	α-SMA, SM22a, calponin, osteopontin	Promote	^182^
Atrial fibrillation	LncRNA	LncRNA MIAT	miR-133a-3p	Promote	^214^
Atherosclerosis	CircRNA	Circ-SATB2, CircRNA-0044073,CircR-284, Circ_RUSC2	mir-939, mir-107, mir-221, mir-661	Promote	^185–188^
Myocardial infarction	CircRNA	CircRNAcdr1as, MFACR, Circ_Nfix	mir-7a, miR-652-3p, miR-214	Promote	^189–192^
Myocardial infarction	CircRNA	Circ-Ttc3	miR-15b-5p	Inhibit	^191^
Cardiac fibroblasts proliferation and migration	CircRNA	CircSlc8a1, CircNFIB	miR-133a, miR-433	Inhibit	^193,196^
Cardiac fibroblasts proliferation and migration	CircRNA	CircRNA_010567, CircHIPK3,	mir-141, miR-29b-3p,	Promote	^194,195^
Ischemic heart disease	CircRNA	Circ-ncx1,	miR-133a-3p	Promote	^197^
Ischemic heart disease	CircRNA	Circ ACR	Pink1/ FAM65B	Inhibit	^198^
Heart failure	CircRNA	Mmu_circ_0005019	miR-499-5p	Inhibit	^200^
Heart failure	CircRNA	circRNA CDYL	miR-4793-5p	Inhibit	^201^
Heart failure	CircRNA	circRNA 010567	TGFβ1	Promote	^202^
Heart failure	CircRNA	Circ_LAS1L	miR-125b	Inhibit	^203^
Heart failure	CircRNA	CircRNA_000203	miRNA-26B-5p, miRNA-140-3p	Promote	^204^
Heart failure	CircRNA	CircFndc3b	Fused	Inhibit	^205^
Heart failure	CircRNA	CirRNA ACAP2	miRNA-29	Promote	^206^
Vascular calcification	CircRNA	CDR1as	miRNA-7-5p	Promote	^207^
Vascular calcification	CircRNA	CircRNA TGFBR2	miR-25-3p	Inhibit	^208^
Vascular calcification	CircRNA	CircRNA-vgll3	miRNA-326-5p	Promote	^209^
Hypertension	CircRNA	CircACTA2	miR-548F-5p	Promote	^210^

*SIRT1* Sirtuin1, *ABCA1* ATP-binding cassette transporter A1 gene, *MMP2* matrix metalloproteinase 2, *STAT3* signal transducer and activator of transcription 3, *SM22a* smooth muscle 22 alpha, *α-SMA* alpha-smooth muscle actin, *TGFβ1* transforming growth factor-β1

#### Coronary heart disease and acute coronary syndrome

At present, miRNA has been implicated in the development of coronary heart disease and acute coronary syndrome. One study reported that miRNA-SNP rs41291957 increased the expression of miRNA-143 and miRNA-145 in human coronary smooth muscle cells and modulated the phenotypic conversion of VSMCs. The study suggested that miRNA-SNP rs41291957 can be regarded as a important gene of assessing coronary heart disease risk and prognosis.^118^ Jiang et al.^119^ reported that inhibition of mir-1 not only reduced the inflammatory response of endothelial cells in vitro but also alleviated the occurrence of atherosclerosis.^119^ In addition, miRNA-106a-3p and miRNA-342-5p also have anti-atherosclerotic effects on endothelial cells,^120,121^ whereas miRNA-92a promotes the occurrence of atherosclerosis.^122^ Some studies have shown that miRNA-125b, miRNA-21a-5p, miRNA-25-3p, and miRNA-144 have protective effects on cardiac myocytes, and can be used as potential therapeutic targets for AMI in the future.^123–126^ Ling et al.^127,128^ found that serum levels of exosome miRNA-122-5P and miRNA-126 were positively correlated with coronary artery stenosis in patients with unstable angina and AMI. Therefore, studies have demonstrated that serum exosome miRNA-21, miRNA-122-5p, and miRNA-126 were novel biomarkers for the diagnosis of the acute coronary syndrome.^127,128^ Studies demonstrated that miRNA-590-3p and miRNA-199a-3p could promote the proliferation of myocardial cells in the mice infarct area. It implied that targeted miRNA-590-3p and miRNA-199a-3p treatment could restore the function of myocardial cells after myocardial infarction.^129^

#### Heart failure

According to recent studies, exosome miRNAs play an important role in myocardial remodeling and heart failure.^130^ Wu et al.^131^ observed that elevated serum exosome miRNA-92b-5p levels in patients with acute heart failure were negatively correlated with left ventricular ejection fraction. Serum exosome miRNA-92b-5p can be used as a biomarker of heart failure with reduced ejection fraction.^131^ Wang et al.^132^ reported downregulation of miRNA-425 and miRNA-744 levels in angiotensin-treated cardiac fibroblasts (CFs). miRNA-425 and miRNA-744 inhibited angiotensin-induced collagen and cellulose synthesis, reduced CFs activation, and improved cardiac remodeling by targeting TGF-β. Therefore, miRNA-425 and miRNA-744 were expected to be therapeutic targets and heart failure diagnostic markers for reversing cardiac remodeling. In conclusion, exosome miRNAs are looked forward to being a new tool for the diagnosis and treatment of heart failure.^132^

#### Vascular calcification

miRNA is strongly associated with the occurrence and development of vascular calcification. Xu et al.^133^ established vascular calcification animal models in vitro and in vivo. They found that miRNA-30b was a potential endogenous regulatory factor of vascular calcification, which had a protective effect on calcification. The main mechanism is that miRNA-30b increases MMPs and autophagy in VSMCs by inhibiting mTOR signaling pathway, maintains mitochondrial homeostasis, and attenuates the phenotypic transformation of VSMCs to osteogenic phenotype.^133^ In addition, in β-glycerophosphorate-treated VSMCs, miRNA-30b reduced the VSMC calcification occurrence by targeting to inhibit SOX9, decreasing the activation of bone morphogenetic protein 2 (BMP2) and preventing osteoblast differentiation.^134^ A clinical study showed that plasma miRNA-204 levels were significantly lower in patients with coronary artery calcification than in controls. Plasma miRNA-204 level was significantly and independently correlated with coronary artery calcification. Therefore, plasma miRNA-204 level can be used as a predictor to evaluate coronary artery calcification severity.^135^ However, Wang et al.^136^ reported that miRNA-128‐3p promoted Wnt‐1, β‐catenin, GSK‐3β, and Bax expression by downregulating Islet1 (ISL1) through activation of the Wnt pathway, thereby exacerbating cardiovascular calcification in type 2 diabetic rats.^136^ Chen et al.^137^ showed that miRNA-19A-3p inhibited HDAC4 expression, upregulated Runx2 and osteocalcin levels, and enhanced ALP activity by targeting the 3’UTR of HDAC4. Thus promoting human mesenchymal stem cells (hMSCs) osteogenic differentiation and calcification.^137^

#### Hypertension

miRNA can be an important factor for regulating the pathogenesis of pulmonary hypertension and hypertension. Sind et al.^138^ demonstrated that miRNA-181A-5p and miRNA-324-5p could reduce pulmonary vascular remodeling to resist the occurrence of pulmonary hypertension.^138^ Upregulation of miRNA-34C-5p, miRNA-449b, miRNA-571, miRNA-765, miRNA-483-3p, miRNA-143/145, miRNA-21, miRNA-126, miRNA-196a, miRNA-132, miRNA-212, and miRNA-451 may result RAS imbalance, which raises blood pressure.^139^ Study found that cyclic adenosine phosphate and sex hormones could stimulate the expression of renin mRNA and the secretion of renin proprotein in primary trophoblast cells. After transfection of miRNA-181A-5p and miRNA-663 into trophoblast cells, the expression of renin mRNA and the production of renin proprotein were declined. Thus reducing renin activity and preventing it from lysing angiotensinogen to produce Ang I. In the end, it inhibited RAS response and resulted in decreased blood pressure.^140^

### The role of lncRNAs in cardiovascular disease

LncRNAs is a double-edged sword in cardiovascular disease progression, being both a positive regulator and a negative regulator (Table 4). Studies have shown that Linc1405, LncRNA PANCR, LncRNA Handdown (Hdn) reprograms cardiac fibroblasts into cardiomyocytes, activates cardiomyocyte differentiation, and participates in cardiac development.^141–144^ LncRNA CAREL inhibits cardiac regeneration and differentiation by targeting miRNA-296.^145^ LncRNA NR_045363 and SIRT1 antisense LncRNA activate cardiac regeneration by targeting miRNA-216a and SIRT1 mRNA, respectively.^146,147^ LncRNA CRRL and LncRNA AZIN2-sv regulate the proliferation of cardiomyocytes by sponges miRNA-199a-3p and miRNA-214.^148,149^ REN et al.^150^ found that compared with patients without any obvious complications type II diabetic cardiovascular disease and healthy controls, plasma lncRNA-SRA levels in patients with type II diabetic cardiovascular disease were significantly reduced. The mechanism may be that LncRNA-SRA participates in the regulation of VSMCs proliferation, thus regulating the occurrence and development of cardiovascular diseases.^150^ Chen et al.^151^ first revealed the expression profile of lncRNA in a chronic intermittent hypoxia rat model through lncRNA chip experiment and bioinformatics analysis, providing a new idea for exploring the pathogenesis of cardiovascular diseases induced by obstructive sleep apnea.^151^ Furthermore, the potential value of lncRNAs as diagnostic biomarkers has been widely explored. LncRNA SENCR has certain value in the diagnosis of early-onset coronary artery disease (CAD).^152^ LncRNA DKFZP434I0714 can be used as a biomarker to predict cardiovascular adverse events in uremia patients.^153^

#### Atherosclerosis

LncRNAs play a key role in atherosclerosis. LncRNAs mainly regulate atherosclerosis occurrence and progression by affecting inflammatory response, apoptosis and autophagy of vascular endothelial cells, foam cells formation, lipid metabolism and other mechanisms. One study showed that LncRNA ANRIL could be used as a biomarker of atherosclerosis.^154,155^ LncRNA LEENE can enhance the anti-inflammatory ability of endothelial cells.^156^ LincRNA-p21 and smooth muscle-induced lncRNA enhances replication (SMILR) affect the development of atherosclerosis by inducing cell apoptosis and regulating cell proliferation, respectively.^157,158^ In addition, LncRNA Mexis overexpression has been reported to promote cholesterol efflux by increasing ATP-binding cassette transporter A1 gene (ABCA1) expression, thereby reducing the probability of atherosclerosis in mice.^159^ Hu et al.^160^ reported that lncRNA, Nexilin F-actin-binding protein antisense RNA 1 (NEXN-AS1), regulated the expression of actin-binding protein NEXN. Expression microarray analysis showed that the expressions of NEXN-AS1 and NEXN were both decreased in human atherosclerotic plaques. Downregulation of NEXN-AS1 and NEXN expression can enhance the activity of TLR-4 oligomer and NF-κB, upregulate the expression of endothelial cell adhesion molecules and inflammatory factors, and increase the number of macrophages in atherosclerotic lesions, thereby promoting the development of atherosclerosis. These findings suggest that NEXN- AS1 and NEXN have a protective effect on atherosclerosis and may serve as potential therapeutic targets for atherosclerotic diseases in future.^160^

#### Myocardial infarction

LncRNAs are not only early indicators of myocardial infarction,^161,162^ but also act as a crucial element in the pathogenesis of myocardial infarction by controlling autophagy, apoptosis and other processes. LncRNA APF, lncRNA CAIF, and lncRNA Mirf affect the injury of myocardial infarction by regulating cardiac autophagy.^163–165^ Meanwhile, lncRNA Gpr19 inhibition and lncRNA UCA1 overexpression blocked apoptosis by miRNA-324-5p/Mtfr1 axis and miRNA-143/MDM2/p53 axis, respectively, to alleviate myocardial injury after myocardial infarction.^166,167^ In addition, lncRNA CPR, MALAT1, lncRNA AK139128 participate in cardiac repair and cardiac function development after myocardial infarction by regulating cell proliferation.^168–171^ These results suggest that lncRNAs may be potential therapeutic targets for myocardial infarction.

#### Heart failure

In recent years, increasing evidence has accumulated for lncRNA in heart failure progress. Studies have demonstrated that lncRNA LIPCAR, lncRNA COL1A1, and lncRNA H19 can be regarded as biomarkers to predict and evaluate the risk of heart failure.^172–174^ Piccoli et al.^175^ reported that lncRNA Meg3 was downregulated in late cardiac remodeling. Meg3 inhibition downregulated expression and activity of MMP2, leading to disminish cardiac fibrosis and hypertrophy.^175^ Another type of myocardial fibroblast enriched lncRNA Whisper (Wisper 2 superenhance-associated RNA) is primarily used to regulate myocardial fibrosis after injury. The results shows that silencing Wisper could alleviate myocardial infarction-induced fibrosis and cardiac dysfunction through upregulating myocardial fibroblasts Col3a1, Fn1, Tgfb2, and aSma expression.^176^ These results provide new insights into the pathogenesis of heart failure and contribute to enhance diagnostic performance and treatment strategies for heart failure, thereby improving the long-term prognosis of heart failure.

#### Vascular calcification

LncRNA is of the utmost importance in the development of vascular calcification. Yu et al.^177^ found that LncRNATUG1 was significantly expressed in human aortic valve and primary valvular interstitial cells (VICs). LncRNATUG1 activation downregulated the expression of miRNA-204-5p by sponging and reversed the inhibition of Runx2 by LncRNATUG1 short hairpin RNA (shRNA). Thereby, the levels of osteoblastic-specific proteins (such as osteocalcin, OPN, and osterix) were upregulated to promote calcific aortic valve disease osteoblastic differentiation. Therefore, LncRNATUG1 can serve as a positive regulator of osteogenic differentiation in the calcific aortic valve disease pathogenesis.^177^ Jeong et al.^178^ reported that after knockdown Lrrc75a-as1, calcium deposition increased, while its overexpression inhibited calcium deposition in A10 cells. Lrrc75a-as1 overexpression reduced the expression levels of osteogenic factors Runx2, msh homeobox 2 and BMP2 in VSMCs by regulating transcription factors SRF, CREB1, and STAT3. Finally, it slowed down vascular calcification process. Taken together, studies have indicated that Lrrc75a-as1 is a negative regulator of vascular calcification.^178^ Huang et al.^179^ reported that lncRNA-SNHG29 expression was downregulated and miRNA-200b-3p expression was upregulated in vitro calcification model. LncRNA-SNHG29 could activate the α-Klotho/FGFR1/FGF23 axis in VSMCs by downregulating miRNA-200b-3p, upregulate the α-Klotho target of miRNA-200b-3p, and inhibit the Wnt/β-catenin signaling pathway. Meanwhile, it also promoted FGFR1 and FGF23 expression, which significantly inhibited osteogenic factors (Runx2 and BMP2) and prevented VSMC calcification. These results suggest that LncRNA-SNHG29 can be a novel therapeutic target for vascular calcification-related diseases.^179^ A study revealed that Bhlhe40 overexpression inhibited the lncRNA-ES3 expression by binding to the promoter region of lncRNA-ES3 gene (LINC00458), and then upregulated the expressions of miRNA-95-5p, miRNA-6776-5p, miRNA-3620-5p, and miRNA-4747-5p, decreased ALP activity and secretion of osteocalcin, which alleviated human aortic VSMCs calcification induced by high glucose.^180^

#### Hypertension

LncRNA has been implicated in the development of hypertension. Yang et al.^181^ reported that HAS2-AS1 (an extracellular matrix-associated lncRNA) and C/EBPβ were highly expressed in hypoxic HFL-1 cells. C/EBPβ bound to the promoter region of HAS2-AS1 to activate its transcription and promoted the inflammatory response of HFL-1 cells. Downregulation of HAS2-AS1 expression inhibited the HFL-1 cells proliferation, migration, and inflammatory response. Thus, the study reveals that HAS2-AS1 may be involved in the pathophysiology of hypoxic pulmonary hypertension.^181^ Specific siRNA was used to knockdown MRAK048635_P1 from VSMCs isolated from the thoracic aorta of hypertensive rats. It was found that the downregulation of MRAK048635_P1 could stimulate the proliferation and migration of VSMCs and induce the transformation from contractile to the secretory phenotype of VSMCs. It suggests that decreased expression of LncRNA MRAK048635_P1 could be used as an important factor for vascular remodeling in hypertension.^182^

### The role of circRNAs in cardiovascular disease

#### Coronary heart disease and myocardial infarction

More and more attention has been paid to the regulation of cirRNAs on coronary heart disease and myocardial infarction. Lin and Pan et al.^183,184^ found that cirRNAs might regard as a potential clinical marker for the diagnosis of coronary heart disease through high-throughput technology and competitive endogenous ceRNA chip analysis.^183,184^ CirRNA-SATB2, cirRNA-0044073, cirRNA-284, and cirRNA_RUSC2 participate in the development of atherosclerosis by targeting miRNA-939, miRNA-107, miRNA-221, and miRNA-661 to regulate VSMCs proliferation and migration, respectively.^185–188^ CirRNA cdr1as, MFACR, and Cir_Nfix play key roles in promoting cardiac regeneration repair and apoptosis by targeting miRNA-7a, miRNA-652-3p, and miRNA-214, respectively, providing new evidence for further research on myocardial infarction. Circ-Ttc3 protects myocardial infarction-induced myocardial apoptosis by inhibiting the activity of miRNA-15b-5p. CircNfix regulates the Gsk3b signaling pathway through miRNA-214. The downregulation of circNfix expression can ameliorate myocardial infarction.^189–192^ CircSlc8a1, CircRNA_010567, CircHIPK3, and CircNFIB regulate the proliferation and migration of cardiac fibroblasts through sponges miRNA-133a, miRNA-141, miRNA-29b-3p, and miRNA-433. Thus changing the cardiac structure and the development of cardiac dysfunction.^193–196^ CircNCX1 promotes myocardial apoptosis and myocardial ischemia–reperfusion injury through competitively binds miRNA-133A-3p. Circ ACR attenuates myocardial ischemia/reperfusion injury by suppressing autophagy.^197,198^

#### Heart failure

CirRNAs are deemed as novel regulatory genes in cardiomyocyte hypertrophy, fibrosis, autophagy, and apoptosis, which are involved in the development of heart failure.^199^ Mmu_circ_0005019 regulates the expression of its target gene Kcnn3 by targeting miRNA-499-5p, thereby inhibiting cardiac fibrosis and reversing electrical remodeling of cardiac myocytes.^200^ In addition, circNfix inhibit the development of heart failure by regulating the proliferation of cardiomyocytes after myocardial infarction. CirRNA CDYL overexpression can promote myocardial cell proliferation in vitro by targeting miRNA-4793-5p.^201^ Therefore, circNfix and CDYL have the potential to be used as key modulatory factors to ameliorate the prognosis of myocardial infarction and delay the progression of heart failure. Furthermore, reduced cirRNA 010567 expression alleviates myocardial fibrosis by blocking the TGFβ1 signaling pathway, thereby improving cardiac function.^202^ Circ_LAS1L inhibits cardiac fibroblasts proliferation by increasing the expression of SFRP5 through sponge miRNA-125b.^203^ Therefore, cirRNA 010567 and circ_LAS1L slow the progression of heart failure by reducing myocardial fibrosis and preventing ventricular remodeling. Nevertheless, cirRNA_000203 aggravate cardiac hypertrophy through sponge miRNA-26b-5p and miRNA-140-3p to aggravate cardiac hypertrophy, which may increase the risk of heart failure.^204^ CircFndc3b overexpression increased angiogenic activity and reduced cardiomyocytes and endothelial cells apoptosis by interacting with RNA binding protein Fused. Therefore, these findings suggest that CircFndc3b can ameliorate cardiac remodeling and cardiac function after myocardial infarction.^205^ CirRNA ACAP2 induces myocardial apoptosis after myocardial infarction by binding to miRNA-29.^206^ Thus, cirRNAs are closely relevant to heart failure development.

#### Vascular calcification and hypertension

The role of the cirRNA in the development of vascular calcification and hypertension will be discussed. CDR1as is an important cirRNA. Recently, a study showed that CDR1as might act as a molecular sponge for miRNA-7-5p. Under hypoxia induction, the expression of CDR1as and its target genes CAMK2D and CNN3 were upregulated, while the expression of miRNA-7-5p was downregulated. The co-transfection of siCDR1as and miRNA-7-5p antagonist can promote human pulmonary artery smooth muscle cell (HPASMC) mineralization. It suggests that the CDR1as regulatory role in HPASMC calcification may be related to the inhibition of miRNA-7-5p function. Overexpression of CAMK2D and CNN3 enhances HPASMC mineralization under hypoxia induced by CDR1as and miRNA-7-5p agonists. In conclusion, the study demonstrated that in hypoxic HPASMCs, CDR1as upregulated CAMK2D and CNN3 expression through sponge miRNA-7-5p, accelerating HPASMC osteoblasts differentiation and calcification.^207^ Yu et al.^208^ reported that TGFBR2 expression was downregulated and miRNA-25-3p expression was upregulated in osteogenic induced aortic VIC. TGFBR2 sponge miRNA-25-3p regulates TWIST1 expression in osteogenic induced VIC. Overexpression of miRNA-25-3p or TWIST1 knockdown increases osteoblast markers Runx2 and OPN expression and ALP activity, leading to calcium nodular formation. Therefore, overexpression TGFBR2 can inhibit VIC osteoblasts differentiation and calcification by interacting with miRNA-25-3p and TWIST1.^208^ Research data showed that cirRNA-vgll3 originated from the vgll3 site acted as a miRNA-326-5p sponge. CircRNA-vgll3 overexpression attenuates miRNA-326-5p-mediated integrinα5 (Itga5) inhibition by targeting miRNA-326-5p. The mRNA expression levels of osteogenic marker genes in adipose-derived mesenchymal stem cells including Runx2, osterix, OPN, osteocalcin and BMP2 were markedly enhanced. Thus cirRNA-vgll3 overexpression significantly accelerated adipose-derived mesenchymal stem cells osteogenic differentiation.^209^ In addition, Sun et al.^210^ demonstrated that circACTA2 interacts with miRNA-548F-5p targeting α-SMA mRNA3'-UTR, which alleviated the inhibition of α-smooth muscle actin(α-SMA) expression by miRNA-548F-5P. Thereby upregulating the expression of α-SMA, promoting the contraction of VSMCs and regulating vascular tension, and participating in the occurrence of hypertension.^210^ These results suggest that circRNAs may be potential diagnostic biomarkers for vascular calcification and hypertension.

In conclusion, these findings provide a new perspective for the study of cirRNA in cardiovascular disease (Table 4). After understanding the regulation of epigenetics in different cardiovascular diseases. To sum up, the following comments mainly describe the related drugs and potential targets of epigenetic therapy for cardiovascular disease. It will create more possibilities for the future clinical application of epigenetic drugs for cardiovascular disease.

### The role of epigenetics regulation in other cardiovascular diseases

In recent years, epigenetics has been gradually studied in other cardiovascular diseases. Bahado-singh et al.^211^ found 165 significantly differentially methylated CpG loci in the tetralogy of Fallot cases. Among 165 CpG sites with differential methylation, cg05273049, cg02540011, cg08404201, and cg00687252 had the highest predictive accuracy. These methylation sites can be used as biomarkers to predict tetralogy of Fallot with good accuracy. Therefore, this study shows that there is a significant correlation between DNA methylation changes in the placenta and tetralogy of Fallot.^211^ As an important member of histone lysine methyltransferases, the SET domain (SETD) family plays a key role in histone modification. SETD1B activates Notch signaling by upregulating the level of H3K4me3 in endothelial cells and exacerbates endothelial inflammation and apoptosis. These findings suggest that SETD1B-based epigenetic reprogramming may potentially improve the course and prognosis of endothelial inflammation-related cardiovascular disease.^212^ In one study, atrial fibrillation was associated with decreased HDAC2 expression and increased neuron-restrictive silencer factor (NRSF) expression. HDAC2 gene knockdown and increased NRSF expression resulted in decreased KCNJ2 K+ ion channel expression and prolonged action potential duration in neonatal rat cardiomyocytes. These new insights into the mechanisms of epigenetic remodeling may provide the theoretical basis for the treatment of atrial fibrillation.^213^ Yao et al.^214^ found that the expression of noncoding RNA myocardial infarction-associated transcript (MIAT) was significantly increased and miRNA-133A-3p was significantly decreased in the rat model induced by atrial fibrillation. MIAT knockdown significantly alleviates atrial fibrillation and reduces atrial fibrillation-induced atrial fibrosis by targeting miRNA-133A-3p and inhibiting fibrosis-related gene expression of collagen I, collagen III, connective tissue growth factor, and TGF-β1.^214^ Costantino et al.^215^ demonstrated that in diet-induced obese mouse hearts, AP-1 transcription factor directly bound to the PPARγpromoter, which resulted in activation of PPARγand increased transcription of Fas, Cd36, Lpl and Plin5. Thereby it promoted lipid accumulation, cardiac dysfunction, and ultimately led to metabolic cardiomyopathy. JunD is a direct target of miRNA-494-3p. miRNA-494-3p overexpression can reduce lipid accumulation and obesity-related metabolic cardiomyopathy by inhibiting JunD/PPARγ signaling pathway. These findings open new therapeutic strategies for metabolic cardiomyopathy and left ventricular dysfunction in obese patients.^215^ Although there is still much unknown about the mechanism of epigenetics in other cardiovascular diseases, its important significance in biology has been highlighted. In future, we look forward to exploring more new breakthroughs in epigenetics in other cardiovascular diseases.

### Epigenetic therapy for cardiovascular disease

#### DNA methylation

**Fig. 5:**
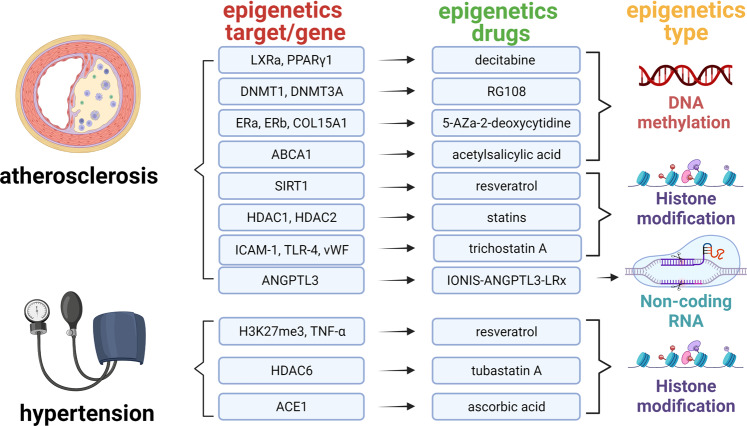
Epigenetics-related targets and drugs in atherosclerosis and hypertension. This figure was created with the aid of Biorender (https://biorender.com/). LXRa, liver X receptor a, PPARγ1 peroxisome proliferator-activated receptorγ1, DNMTs DNA methyltransferases, ER estrogen receptor, COL15A1 collagen, type XV, alpha 1, ABCA1 ATP-binding cassette transporter A1 gene, SIRT1 sirtuin1, HDAC histone deacetylase, ICAM-1 intercellular adhesion molecule-1, TLR-4 toll-like receptor-4, vWF von Wilebrand factor, ANGPTL3 angiopoietin-like 3, H3K27me3 trimethylated histone 3 lysine 27, TNF-a tumor necrosis factor (TNF)-a, ACE1 angiotensin-converting enzyme 1

**Fig. 6:**
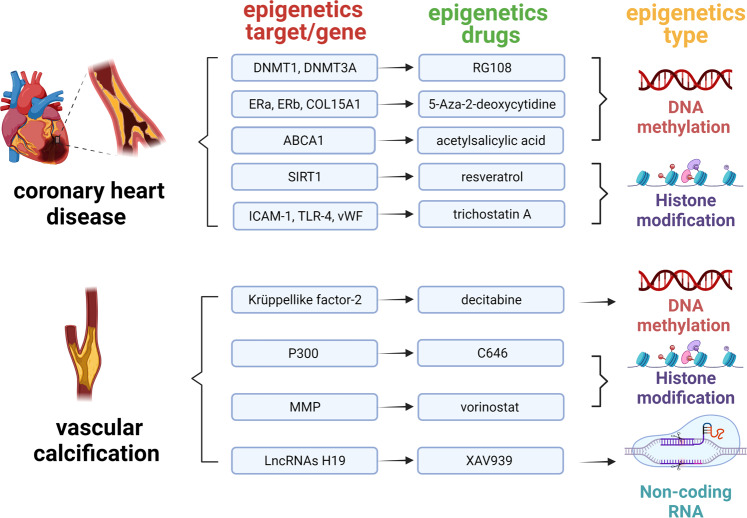
Epigenetics-related targets and drugs in coronary heart disease and vascular calcification. This figure was created with the aid of Biorender (https://biorender.com/). DNMTs DNA methyltransferases, ER estrogen receptor, COL15A1 collagen, type XV, alpha 1, ABCA1 ATP-binding cassette transporter A1 gene, SIRT1 sirtuin1, HDAC histone deacetylase, ICAM-1 intercellular adhesion molecule-1, TLR-4 toll-like receptor-4, vWF von Wilebrand factor, MMP matrix metalloproteinase

**Fig. 7:**
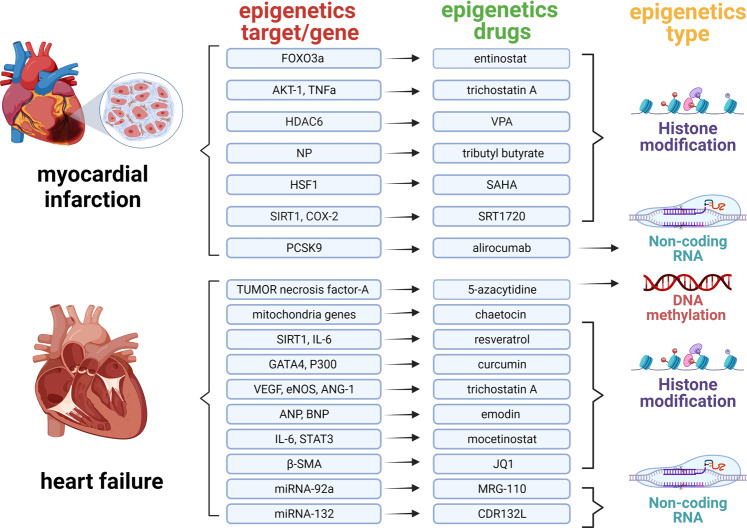
Epigenetics-related targets and drugs in myocardial infarction and heart failure. This figure was created with the aid of Biorender (https://biorender.com/). FOXO3a forkhead box O3a, AKT-1 protein kinase B-1, TNF-a tumor necrosis factor (TNF)-a, HDAC histone deacetylase, NP natriuretic peptide, HSF1 heat-shock transcription factor 1, SIRT1 sirtuin1, COX-2 cyclooxygenase-2, PCSK9 proprotein convertase subtilisin-kexin type 9, IL-6 interleukin 6, VEGF vascular endothelial growth factor, eNOS endothelial nitric oxide synthase, ANG-1 angiopoietin-1, ANP atrial natriuretic peptide, BNP brain natriuretic peptide, STAT3 signal transducer and activator of transcription 3, β-SMA β-smooth muscle actin

DNA methyltransferase inhibitors or related drugs play a role in the treatment of cardiovascular diseases such as coronary heart disease and heart failure by regulating target genes methylation status and expression level (Figs. 5–7). Currently, though, research on DNA methylation as a treatment for cardiovascular disease is still in the development stage. However, because DNA methylation changes are reversible, which offers an optimistic prospect for the treatment of the disease (Table 5).

**Table 5:** Potential epigenetic drugs for the treatment of cardiovascular diseases

Epigenetic classification	Drugs	Type	Target gene	Clinical trial stage	Diseases	References
DNA methylation	Decitabine	DNA methyltransferase inhibitor	LXRa, PPARγ1	–	Atherosclerosis	^216,217^
DNA methylation	RG108	DNA methyltransferase inhibitor	DNMT1, DNMT3a	–	Atherosclerosis coronary heart disease, heart failure	^218,219^
DNA methylation	5-AZa-2-deoxycytidine	DNA methyltransferase inhibitor	ERa, ERb, COL15A1	–	Atherosclerosis coronary heart disease, heart failure	^220,221^
DNA methylation	Acetylsalicylic acid	DNA methyltransferase inhibitor	ABCA1	Phase 3	Atherosclerosis coronary heart disease	^223^
DNA methylation	5-azacytidine	DNA methyltransferase inhibitor	TUMOR necrosis factor-A	–	Heart failure	^225–227^
DNA methylation	Decitabine	DNA methyltransferase inhibitor	Krüppellike factor-2	–	Calcification	^229^
Histone methylation	Chaetocin	Histone H3K9 methyltransferase inhibitor	Mitochondria-related genes	–	Chronic heart failure	^234^
Histone methylation	Resveratrol	Histone methylation-related drugs	H3K27me3	Phase 2	Hypertension	^235^
Histone acetylation	Resveratrol	sirtuin 1 natural agonist	sirtuin 1	Phase 2	Atherosclerosis coronary heart disease	^236^
Histone acetylation	Statins	HDAC inhibitor	HDAC1, HDAC2	Phase 4	Atherosclerosis	^237^
Histone acetylation	Trichostatin A	HDAC inhibitor	ICAM-1, surface TLR-4, vWF	–	Atherosclerosis coronary heart disease	^238^
Histone acetylation	Entinostat	Class I HDAC inhibitor	nuclear FOXO3a transcription factor	–	Myocardial infarction	^239,240^
Histone acetylation	Trichostatin A	HDAC inhibitor	AKT-1, TNF-a	–	Myocardial infarction	^241–246^
Histone acetylation	Valproic acid (VPA), tributyl butyrate, suberoylanilide hydroxamic acid (SAHA)	HDAC inhibitor	HDAC6, NP, HSF1	–	Myocardial infarction	^247–252^
Histone acetylation	Resveratrol	sirtuin 1 natural agonist	sirtuin 1, peroxisome proliferator-activated receptor γ coactivator -1α	–	Ischemia–reperfusion injury	^253–256^
Histone acetylation	SRT1720	sirtuin 1 activator	sirtuin 1, COX-2	–	Myocardial infarction	^257^
Histone acetylation	sildenafil, adiponectin	Phosphodiesterase 5 inhibitors	sirtuin 1	Phase 4	ischemia–reperfusion injury	^258,259^
Histone acetylation	tadalafil	Phosphodiesterase 5 inhibitors	sirtuin 1, PGC-1α, eNOS, Akt, AMPK	Phase 3	Diabetic heart dysfunction	^260^
Histone acetylation	Apicidin	Class I HDAC inhibitor	tuberous sclerosis complex 2	–	Cardiac hypertrophy	^262^
Histone acetylation	Resveratrol	sirtuin 1 natural agonist	sirtuin 1, IL-6	Phase 2	Heart failure	^263^
Histone acetylation	Curcumin	HAT inhibitor	GATA4, P300	–	Heart failure	^266^
Histone acetylation	Trichostatin A	Class I and II HDACs inhibitors	VEGF, eNOS, Ang-1	–	Heart failure	^267^
Histone acetylation	API-D	Class I HDAC inhibitors	Sk Act	–	Heart failure	^268^
Histone acetylation	Emodin	HDAC inhibitors	ANP, BNP	–	Heart failure	^272^
Histone acetylation	MPT0E014	HDAC inhibitors	Peroxisome proliferator-activated receptor, IL-6, p22, SMAD2/3	–	Heart failure	^273^
Histone acetylation	Mocetinostat	Class I HDAC inhibitors	IL-6, STAT3	–	Heart failure	^274,275^
Histone acetylation	Valproic acid	HDAC inhibitors	Mineralocorticoid receptor	–	Heart failure	^276^
	JQ1	bromodomain-containing protein 4 inhibitor	β-SMA	–	Heart failure	^278,279^
Histone acetylation	C646	HAT inhibitor	P300	–	Vascular calcification	^281^
Histone acetylation	Vorinostat	HDAC inhibitor	MMPs	–	Vascular calcification	^283^
Histone acetylation	Resveratrol	sirtuin 1 inhibitor	TNF-α	–	Pulmonary hypertension	^285^
Histone acetylation	Tubastatin A	HDAC6 inhibitor	HDAC6	–	Hypertension	^114^
Histone acetylation	Ascorbic acid	HDAC inhibitor	ACE1	Phase 1	Hypertension	^287^
Noncoding RNAs	Inclisiran	Long-acting RNA interference therapeutic agent	PCSK9	Phase 3	Familial hypercholesterolemia	^51–55^
Noncoding RNAs	AKCEA-APOCIII-L_Rx_	N-acetylgalactosamine-conjugated antisense oligonucleotide	apolipoprotein C-III	Phase 2	Hypertriglyceridemia	^60^
Noncoding RNAs	IONIS-ANGPTL3-LRx	Antisense oligonucleotides	ANGPTL3	–	Atherosclerosis	^56^
Noncoding RNAs	SNHG12	lncRNA	DNA-PK	–	Atherosclerosis	^288^
Noncoding RNAs	volanesorsen	2’-O-methoxyethyl (2’-MOE) chimeric antisense therapeutic oligonucleotide	APOC3	Phase 3	Hypertriglyceridemia	^289,290^
Noncoding RNAs	IONIS-APO(a)-LRx	Antisense oligonucleotides	apolipoprotein(a)	Phase 2	Elevated Lipoprotein(a)	^49^
Noncoding RNAs	Alirocumab	PCSK9 antibody	PCSK9	Phase 4	Acute coronary syndrome	^57^
Noncoding RNAs	lncRNA MIAT	lncRNA	miR-150-5p, VEGF	–	Myocardial infarction	^295^
Noncoding RNAs	circRNA MFACR	circRNA	miRNA-125b	–	Myocardial infarction	^296^
Noncoding RNAs	MRG-110, CDR132L	Noncoding RNA	miRNA-92a, miRNA-132	Phase 1	Heart failure	^297^
Noncoding RNAs	circ-FOXO3	circRNA	CDK2, cyclin-dependent kinase inhibitor 1	–	Heart failure	^299^
Noncoding RNAs	lncRNA-ANCR	lncRNA	Runx2, BMP2	–	Vascular calcification	^300^
Noncoding RNAs	XAV939	Wnt-β-catenin signaling pathway inhibitor	lncRNAs H19	–	Vascular calcification	^301^

*LXRa* liver X receptor a, *PPARγ1* proliferator-activated receptor γ1, *DNMTs* DNA methyltransferases, *ER* estrogen receptor, *COL15A1* Collagen, type XV, alpha 1, *ABCA1* ATP-binding cassette transporter A1, *H3K27me3* trimethylated histone 3 lysine 27, *HDAC* histone deacetylase, *ICAM-1* intercellular adhesion molecule-1, *TLR-4* Toll-like receptor-4, *vWF* von Wilebrand factor, *FOXO3a* Forkhead box O3a, *TNF-a* tumor necrosis factor (TNF)-a, *NP* natriuretic peptide, *HSF1* heat-shock transcription factor 1, *COX-2* Cyclooxygenase-2, *eNOS* endothelial nitric oxide synthase, *AMPK* Adenosine monophosphate-activated protein kinase, *IL-6* interleukin 6, *VEGF* vascular endothelial growth factor, *Ang-1* angiopoietin-1, *ANP* atrial natriuretic peptide, *BNP* brain natriuretic peptide, *STAT3* signal transducer and activator of transcription 3, *β-SMA* β-smooth muscle actin, *MMPs* matrix metalloproteinases, *ACE1* angiotensin-converting enzyme 1, *PCSK9* proprotein convertase subtilisin-kexin type 9, *ANGPTL3* angiopoietin-like 3, *APOC3* Apolipoprotein C3, *Runx2* runt-related transcription factor 2, *BMP2* Bone morphogenetic protein 2, *DNA-PK* DNA-dependent protein kinase

#### Atherosclerosis and coronary heart disease

Studies have shown that 5-Aza-dC (decitabine) treatment of Ldlr−/− mice can inhibit the migration and adhesion of macrophages to epithelial cells, reduce the infiltration of macrophages into atherosclerotic plaques and the expression of inflammatory genes in macrophages,^216^ thus alleviating atherosclerotic lesions and reducing the development of atherosclerosis.^217^ DNMT inhibitor RG108 plays an important role in atherosclerosis and coronary heart disease by inhibiting DNMT1 and DNMT3a activity.^218,219^ 5-AZa-2-deoxycytidine (DAC) demethylation has been demonstrated to treat atherosclerosis and coronary heart disease by upregulating the expression of normal smooth muscle cells and endothelial cells ERa, ERb and COL15A1.^220,221^ A recent study reported that Cocoa extract improved atherosclerosis and coronary heart disease by inhibiting DNMTs and methylenetetrahydrofolate reductase (MTHFR) gene expression levels in vitro.^222^ In adults with cardiovascular risk factors, Cocoa in combination with statins reduce cholesterol levels and thus exert a protective effect on the cardiovascular system. (ClinicalTrials.-gov identifier: NCT00502047).^222^ High methylation levels of the ABCA1 have been found to be associated with coronary heart disease and aging. Acetylsalicylic acid (ASA) treatment can decrease ABCA1 DNA methylation level, thereby reducing the occurrence of atherosclerosis and coronary heart disease.^223^

#### Heart failure

DNA methylation is closely related to the treatment of heart failure. It is reported that RG108 can reduce the progression of myocardial hypertrophy and myocardial fibrosis by inhibiting DNA methyltransferase.^224^ Studies have shown that DNA methylation inhibitor 5-azacytidine can decrease the harmful effects of tumor necrosis factor-a on SECRA2a expression.^225^ 5-azacytidine may improve cardiac hypertrophy and reduce myocardial fibrosis by inhibiting DNA methyltransferase and blocking the expression of hypertrophic cardiomyopathy genes.^226,227^ Xiao et al.^228^ demonstrated that 5-AZa-2-deoxycytidine reversed the changes of the myocardial proteome in rats by inhibiting DNA methyltransferase, reduced myocardial hypertrophy, improved myocardial contractility, and eliminated the susceptibility to ischemic injury.^228^

#### Vascular calcification

DNA methyltransferase inhibitor can be used as a potential drug to prevent or treat vascular calcification. Tanaka et al.^229^ demonstrated that DNA methyltransferase inhibitor decitabine might enhance Krüppellike factor-2 (KLF2) mRNA and protein expression by demethylation of KLF2. KLF2 overexpression enhanced transcription of IL-10 and TGFβ1 genes, which inhibited osteoclast differentiation and interacts with Runx2 to induce osteogenic differentiation and calcification.^229^

In summary, DNA methylation inhibitor(decitabine), has a therapeutic effect in basic studies of atherosclerosis and calcification. DNMT inhibitor RG108 and 5-AZa-2-deoxycytidine can slow the progression of atherosclerosis, coronary heart disease and heart failure. Therefore, in future, large-scale multi-center clinical trials of DNA methylation inhibitors decitabine, RG108, and 5-AZa-2-deoxycytidine are expected to carry out to verify their efficacy in patients with cardiovascular disease. These are promising drugs for the treatment of cardiovascular disease. The changes of DNA methylation is reversible, and methylation inhibitors can change the methylation status and expression level of some genes, thus bringing into the corresponding biological effects. In terms of drug effects in epigenetic pharmacology, the influence of DNA methylation is being explored, which provides us with a new perspective to understand and treat cardiovascular diseases. At present, the research of DNA methylation-related drugs in the treatment of various cardiovascular diseases is still in the development stage, which needs to be further excavated in a deeper level.

### Histone modifications

According to recent research findings, histone methyltransferase inhibitors or HAT/HDAC inhibitors are still rarely used in clinical treatment of cardiovascular diseases. However, the development of drugs targeting the mechanisms of histone methylation and histone acetylation has achieved some effect in basic experimental research on the treatment of cardiovascular disease (Figs. 5–7). In future, these drugs are looking forward to being more applied in clinical trials, in order to better meet the needs of ameliorating cardiovascular disease patients' symptoms and prognosis (Table 5).

### Histone methylation

Xiao et al.^230^ found that SMYD4 belonged to the lysine methyltransferase family. As a histone methyltransferase, SMYD4 also regulated histone acetylation by interacting with HDAC1. Transcriptome and bioinformatics analysis of smyd4L544Efs*1 and wild-type developing hearts showed that SMYD4 was a critical epigenetic regulator of heart development, involved in the regulation of endoplasmic reticulum mediated protein processing and primary signaling and metabolic pathways during heart development in zebrafish. Therefore, SMYD4 had the potential to be used as a therapeutic target in embryonic development and cardiogenesis.^230^ SUV39H1 was a histone methyltransferase that catalyzes increased methylation of histone 3 lysine 9. Upregulation of SUV39H1 significantly reduced infarct size and myocardial injury after ischemia–reperfusion injury by decreasing the activity of the mitogen-activated protein kinase family and its downstream transcription factor NF-κB. Therefore, SUV39H1 can be regarded as a treatment strategy for ischemia–reperfusion injury in diabetes mellitus.^231^ Weng et al.^232^ reported that IFN-γ treatment enhanced the expression of histone H3K9 trimethyltransferase SUV39H1 in endothelial cells and promoted the recruitment of SUV39H1 to eNOS promoter. SUV39H1 silencing removes IFN-γ inhibition of eNOS by eliminating H3K9Me3 on the eNOS promoter. Therefore, SUV39H1 might be used as a drug target to intervene in endothelial dysfunction.^232^ Ono et al.^233^ demonstrated that cardiac chronic stress could gradually promote intron repeat regions excessive heterochromatinization of genes critical to cardiac pumping function, such as those related to mitochondrial function. Excessive heterochromatinization of mitochondrial gene repeat elements in failing hearts may lead to gene silencing and impair cardiac function. The histone H3K9 methyltransferase inhibitor chaetocin can maintain appropriate chromatin structure and reverse excessive heterochromatinization. Chaetocin improves myocardial hypertrophy by inhibiting H3K9 methyltransferase.^233^ Therefore, chaetocin will be a potential drug for treating chronic heart failure in the future.^234^ In addition, a study reports that resveratrol may exert a therapeutic effect in DOCA salt hypertension through vascular H3K27me3 methylation.^235^

### Histone acetylation

#### Atherosclerosis

Histone acetylation is crucial to the treatment of atherosclerosis. Arunachalam et al.^236^ demonstrated that resveratrol could ameliorate metabolic disorders, atherosclerosis and coronary heart disease by upregulating SIRT1 in endothelial cells.^236^ Furthermore, it has been reported that statins might prevent the loss of HDAC1 and HDAC2 binding to IL-8 (CXCL8) and MCP1 (CCL2) gene promoters, partially restoring the overall activity of HDAC.^236^ Statins decreased oxLDL-associated histone modifications (H3S10 phosphorylation; acetylation of H3K14 and H4K8) and recruitment of CREB-binding proteins 300, NF-KB, and RNA polymerase II, which in turn control atherosclerotic inflammation. In the end, it plays a significant impact on the pathogenesis of atherosclerosis.^237^ In addition, trichostatin A, an HDAC inhibitor, blocks upregulation of uremia environment-induced markers of endothelial dysfunction (intercellular adhesion molecule-1, surface Toll-like receptor-4, von Wilebrand factor) and reactive oxygen species. Thereby trichostatin A could treat atherosclerosis and coronary heart disease.^238^

#### Myocardial infarction and ischemia–reperfusion injury

Histone deacetylase inhibitors are deemed as underlying drugs and strategies for the treatment of myocardial infarction and ischemia–reperfusion injury. Study showed that entinostat (MS-275), a class I-specific HDAC inhibitor, increased the expression of SOD2 and catalase in myocardial mitochondria after ischemia–reperfusion via the nuclear FOXO3A transcription factor. Thus, MS-275 significantly reduced the size of myocardial infarction and improved left ventricular function and tissue vitality. Finally, it protected cardiac systolic function after ischemia–reperfusion.^239,240^ It was found that trichostatin A treatment significantly inhibited the activity of HDAC, increased the phosphorylation of AKT-1, and reduced myocardium and serum TNF-a. Consequently, trichostatin A increased the formation of myocardial cells and cardiac microvessels. Meanwhile, it significantly reduced myocardial infarction size, and generated a protective effect in the heart of patients with myocardial infarction. These results suggest that inhibition of HDAC can maintain cardiac function and attenuate cardiac remodeling by stimulating endogenous cardiac regeneration.^241–246^ In the context of ischemia/reperfusion injury and myocardial infarction, HDAC inhibitors valproic acid, tributyl butyrate, and suberoylanilide hydroxamic acid (SAHA) reduce myocardial infarction size and ventricular remodeling by inhibiting HDAC, and induce increased angiogenic response.^47,247,248^ Studies have demonstrated that long-term use of low doses of SAHA ameliorated cardiac remodeling after infarction. Finally, it resulted in a lasting protective impact on the heart and no toxic effects on the heart.^249–251^ Therefore, HDAC inhibitor SAHA is expected to be tested in clinical trials in order to better serve as a potential treatment drug for myocardial infarction in future. In addition, vorinostat was shown to delay ischemia–reperfusion injury in mice and rabbits, which might be a significant drug for future clinical treatment of ischemia–reperfusion injury.^252^ It is reported that long-term use of resveratrol can activate SIRT1, attenuate FOXO1-related pro-apoptotic signaling pathway, increase peroxisome proliferator-activated receptor γ coactivator-1 (PCC-1) α and mitochondrial biogenesis, improve myocardial function and Ang-II-induced cardiac remodeling in aging mice, thereby protecting myocardial cells from ischemia–reperfusion injury.^253–256^ Studies have shown that SRT1720 up-regulates SIRT1, activates the COX-2 signaling pathway, and decreases oxidative stress and inflammation, thereby alleviating mice vascular endothelial dysfunction and reducing myocardial infarction in SIRT1(+/−) hearts.^257^ Phosphodiesterase 5 inhibitors sildenafil and adiponectin play a protective role on the heart by increasing SIRT1 activity in the myocardium.^258,259^ In a diabetic mouse model, tadalafil was administered to activate SIRT1-PGC-1α activity and eNOS, Akt, AMPK phosphorylation. In the end, it ameliorated myocardial function in diabetic mice.^260^ Therefore, targeting SIRT may be a promising approach for treating cardiovascular disease strategies.

### Heart failure

Histone deacetylase inhibitors are vital for the prevention and treatment of heart failure. Wang et al.^261^ showed that a high-fat diet enhanced SUV39H, decreased SIRT1, inhibited AMPK and CaM kinase II autophagy and phosphorylation, thereby contributing to ventricular hypertrophy and interstitial fibrosis, and resulting in cardiac systolic dysfunction. Mitochondrial aldehyde dehydrogenase (ALDH2) fights high-fat diet-induced cardiac structural and functional abnormalities through mechanisms related to autophagy regulation and promotion of SUV39H-SIRT1-dependent PGC-1α deacetylation. Thus it generates a protective effect on the heart.^261^ Morales et al^262^ demonstrated that inhibition of class I HDACs with apicidin induced the expression of the mTOR inhibitor tuberous sclerosis complex 2 (TSC2), which in turn attenuated cardiac hypertrophy by inhibiting mTOR.^262^ In addition, one study data suggest that resveratrol disminishes IL-6 activation induced by SIRT1 to protect H9c2 cells from Ang-II-induced hypertrophy.^263^

Studies have shown that the P300 HAT inhibitor curcumin significantly reduces LDL levels and increases high-density lipoprotein (HDL) levels in healthy volunteers and patients with atherosclerosis.^264,265^ Curcumin can prevent ventricular hypertrophy and maintain systolic function in heart failure rat models by inhibiting histone acetylation and hypertrophy response transcription factor (GATA4) and decreasing P300 HAT activity by disrupting the P300/GATA4 complex.^266^ In addition, class I and II HDACs inhibitors (trichostatin A) and apicidin derivative, API-D (selective inhibitors of class I HDAC) ameliorated cardiac function in thoracic aortic constriction mice by preventing cardiomyocyte hypertrophy and myocardial fibrosis.^267–269^ HDAC4 inhibitors have been reported to block cardiomyocyte hypertrophy, improve cardiac function and inhibit cardiac remodeling in mice.^270^ Trichostatin A and emodin have good clinical value in the treatment of cardiac hypertrophy and heart failure, through deacetylation of histone.^271,272^ It was found that MPT0E014 generated beneficial impact in heart failure by inhibiting HDAC, increasing cardiac function, and alleviating the effects of heart failure on cardiometabolism and inflammation.^273^ HDAC class I inhibitor Mocetinostat reverses myocardial fibrosis in heart failure by reducing myocardial fibroblast activation and inducing cell cycle arrest/apoptosis.^274,275^ Furthermore, valproic acid treatment can reduce myocardial hypertrophy and fibrosis in rats.^276^ The class II-specific HDAC inhibitor MC1568 inhibits the activity of HDAC4 and HDAC5 in skeletal muscle and heart. Therefore it may have potential clinical significance in the treatment of muscle and heart disease.^277^

The study reveals that the histone acetylation reader BRD4 undergoes stimulus-dependent, genome-wide redistribution in cardiac fibroblasts. It is enriched on a set of enhancers and super-enhancers, and leads to RNA polymerase II activation and downstream target gene expression. Therefore, BRD4 is a central regulator of the phenotype of cardiac fibroblasts promoting fibrosis. The BRD4 inhibitor JQ1 decreases the expression of activation markers and extracellular matrix proteins in cardiac fibroblasts. In addition, JQ1 also inhibites the contractile activity and β-SMA expression of myocardial fibroblasts. Administration of JQ1 improves cardiac function in preestablished heart failure or myocardial infarction mice.^46,278^ Thus, BRD4 inhibitor JQ1 will be used as a drug for the clinical treatment of heart failure and myocardial infarction in future.^279^

#### Vascular calcification

Histone acetylation-related drugs and strategies are of great importance in vascular calcification treatment progress. HAT P300 is a transcription coactivator involved in gene expression regulation and protein acetylation. Gu et al.^280^ reported elevated levels of acetylated histone 3 and 4 in human aortic valve calcification. Inhibitors of HAT P300 inhibited acetylated histone 3 and 4, alleviating aortic valve calcification induced by high calcium/high phosphate treatment in vitro.^280^ In addition, Li et al.^281^ found that C646 (P300 inhibitor) could downregulate the osteocalcin gene and protein expression. P300 inhibition might regulate Klotho expression and weaken osteogenic transdifferentiation and calcification in VICs by downregulating HAT activity. Therefore, P300 inhibition could be a potential therapeutic target for vascular calcification.^281^ HDAC6 is a member of the HDAC IIb subfamily. Fu et al.^282^ demonstrated that the HDAC6 expression in Aortic valve (AoV) tissues of patients with aortic stenosis was significantly decreased. Downregulation of HDAC6 might promote AoV calcification through endoplasmic reticulum stress/activating transcription factor 4 (ATF4) mediated osteogenic pathway. Thus, HDAC6 will be a new target for the prevention and treatment of vascular calcification in the future.^282^ IL-1β-induced inflammatory response is associated with osteoarthritis (OA) and vascular calcification development. The HDAC inhibitor vorinostat inhibits IL-1β-induced MMPs expression by P38 and ERK1/2 phosphorylation, thereby attenuating VSMC osteoblastic differentiation and calcification. Therefore, HDAC inhibitor vorinostat will be expected to be an important drug in the treatment of vascular calcification in the future.^283^

#### Hypertension

Histone deacetylase inhibitors can be used as therapeutic targets for pulmonary hypertension. Boucherat et al.^284^ reported that HDAC6 maintained Ku70 in a low acetylation state, blocked the translocation of Bax to mitochondria, and prevented cell apoptosis. Inhibition of HDAC6 could reduce the proliferation and anti-apoptotic ability of pulmonary smooth muscle cells with pulmonary hypertension. Therefore, HDAC6 deficient mice had some protective effect against pulmonary hypertension caused by chronic hypoxia. Study results suggest that pharmacological inhibition of HDAC6 ameliorates established pulmonary hypertension and will be a potential therapeutic target for pulmonary hypertension in the future.^284^ In addition, resveratrol treatment can also alleviate vascular remodeling and prevent the development of pulmonary hypertension.^285^

Endothelial dysfunction is an important determinant of hypertension and its complications. Therefore, it is of great clinical significance to identify to prevent endothelial dysfunction potential therapeutic targets. New evidence suggests that histone acetylation is closely related to the regulation of endothelial function. SIRT6 is a member of the highly conserved NAD + dependent deacetylase (class III HDAC). SIRT6 has some effects such as increasing the bioavailability of vascular nitric oxide, promoting endothelium-dependent vascular dilation, and reducing endothelial cell permeability. In the experiment of SIRT6 gene knockout hypertensive mice, it was found that the loss of endothel-specific SIRT6 significantly increased blood pressure, aggravated endothelial dysfunction and cardiac renal injury. SIRT6 inhibited transcription and expression of Nkx3.2 by deacetylating histone H3K9. Meanwhile, SIRT6 induced the expression of GATA5, a novel blood pressure regulator, to regulate the GATA5-mediated signaling pathway to prevent endothelial injury, improve endothelial cell permeability, and promote nitric oxide production and endothelium-dependent vasodilation. Thereby ultimately preventing hypertension and its complications. In a word, pharmacological targeting of SIRT6 will be an innovative therapeutic strategy for hypertension patients in future.^286^ Tubastatin A (TubA), a highly selective HDAC6 inhibitor, can significantly improve Ang-II-induced vasoconstriction and elevate blood pressure by inhibiting the expression and activity of Ang-II-induced HDAC6 and reducing the degradation of CSEγ. Therefore, TubA can be used as an effective strategy to prevent the progression of hypertension.^114^ Wang et al.^287^ found that prenatal exposure to lipopolysaccharide could lead to hypertension in young rats. Nevertheless, ascorbic acid could prevent hypertension occurrence and development in offspring of rats prenatal exposed to lipopolysaccharide. Lipopolysaccharide might induce histone H3 acetylation by inhibiting the enrichment of HDAC1 on ACE1 promoter, resulting in increased ACE1 gene expression in rat offspring and promoting hypertension. However, prenatal treatment with ascorbic acid not only disminished oxidative stress but also downregulated ACE1 gene expression through deacetylation of histone H3 in promoter region ACE1, thereby reducing hypertension risk.^287^ In conclusion, these findings provide potential targets for new antihypertensive therapies that could act as early prevention or treatment for hypertension.

Taken together, statins widely used in clinical practice are histone acetylation inhibitors, which have good efficacy in the treatment of atherosclerosis and coronary heart disease in both basic and clinical trials. Resveratrol has a protective effect on atherosclerosis, coronary heart disease, ischemia–reperfusion injury, heart failure, hypertension, and pulmonary hypertension by regulating histone modification-related factors. Trichostatin A and P300 HAT inhibitor curcumin can improve the progression of atherosclerosis, coronary heart disease and heart failure. But it has not been applied in clinic to treat cardiovascular diseases. Histone modification inhibitors resveratrol, Trichostatin A and Curcumin are expected to be widely used in the treatment of various cardiovascular diseases through further clinical studies. At present, although some inhibitors of HDACs have been applied in the clinical treatment of tumors, their clinical application in cardiovascular diseases is relatively little. Because of the broad spectrum of HDACs substrates, these drugs may lead to nonspecific gene activation or suppression, and they can act on either disease target cells or normal cells with side effects. Therefore, it is necessary to further explore the epigenetic regulation mechanism during the occurrence and development of cardiovascular diseases. The development of histone modification-related drugs with specificity and low side effects on cardiovascular diseases will be the direction of our focus and efforts in the future.

### Noncoding RNA

In recent years, increasing evidence has accumulated for noncoding RNAs function in gene regulation and cardiovascular disease pathogenesis (Figs. 5–7). Noncoding RNAs are attractive targets for potential clinical interventions. Currently, the field of nucleotide gene therapy, including antisense oligonucleotide (ASO) and siRNA, is developing rapidly. Analogs or inhibitors of noncoding RNA are easy to synthesize and have low cytotoxicity when transfected in vivo. Therefore, it will be looking forward to being potential treatment drugs for human heart disease. With the exploration of noncoding RNA mechanism in cardiovascular diseases, we believe that the treatment of noncoding RNA in cardiovascular diseases will usher in a new breakthrough (Table 5).

#### Atherosclerosis

Noncoding RNAs may serve as privotal therapeutic targets in atherosclerosis. Inclisiran (ALN-PCSSC) is a long-acting RNA interference (RNAi) therapeutic agent that inhibits the synthesis of proprotein convertase subtilisin-kexin type 9 (PCSK9). PCSK9 is a target for lowering LDL cholesterol. Inclisiran was observed in phase 1–3 clinical trials with a low incidence of adverse events and significantly reduced LDL cholesterol levels. Inclisiran may provide a novel method for low-density lipoprotein cholesterol (LDL-C) reduction and is also a more successful RNA drug for cardiovascular disease treatment.^51–55^ AKCEA-APOCIII-LRx is a liver-targeted N-acetylgalactosamine-coupled antisense oligonucleotide that selectively inhibits the synthesis of apolipoprotein C-III protein, thus treating hypertriglyceridemia.^60^ One study showed that IONIS-ANGPTL3-LRx was an ASO targeting angiopoietin-like 3 (ANGPTL3) mRNA that reduced atherosclerotic lipoprotein levels in mice and humans and delayed the progression of atherosclerosis.^56^ Haemmig et al.^288^ found that lncRNA small nucleolar host gene-12 (SNHG12) was highly expressed in vascular endothelium, but gradually decreased with the progression of the disease. SNHG12 expression was reduced in pig and human atherosclerotic specimens and negatively correlated with DNA damage and markers of aging. DNA-dependent protein kinase (DNA-PK) is an important regulator of DNA damage response. Deletion of SNHG12 decreased the interaction of DNA-PK with their binding partners Ku70 and Ku80 and increased DNA damage. Intravenous administration of SNHG12 alleviated atherosclerosis by protecting the intima from DNA damage and slowing endothelial aging.^288^ Furthermore, volanesorsen was found to treat hypertriglyceridemia by targeting apolipoprotein C3(APOC3).^289,290^ IONIS-APO(a)-LRx was a novel, tolerable and effective therapy for reducing lipoprotein(a) (Lp[a]) concentration. IONIS-APO(a)-LRx effectively decreased plasma Lp(a) and its associated OxPL, LDL-C, and APOB-100 by targeting apolipoprotein(a). At the same time, IONIS-APO(a)-LRx attenuated adhesion promoting of plasma monocytes, thereby reducing the risk of cardiovascular disease in individuals elevated by Lp(a). Clinical trials had been conducted in patients with cardiovascular disease or calcified aortic stenosis with elevated Lp(a) concentration.^49^ In terms of lipid regulation, inhibition of miRNA-33a and miRNA-33b can reduce plasma in LDL-C level and increase plasma HDL cholesterol level without significant adverse reactions. It suggests that targeted inhibition of miRNA-33 plays an important role in the treatment of hyperlipidemia.^45^ In addition, it has been reported that lncRNA RP5-833A20.1 and lncRNA DYN-LRB2-2 can also be used as atherosclerosis therapeutic targets.^291,292^ Studies have shown that circWDR77 can regulate the proliferation and migration of VSMCs through the sponge-mediated miRNA-124/FGF-2. Therefore, circWDR77 can be used as a therapeutic target for atherosclerosis.^293^

#### Myocardial infarction

The role of noncoding RNA and related drugs in the treatment of myocardial infarction have gradually attracted attention. Among patients with acute coronary syndrome treated with high-intensity statins, patients treated with alirocumab had a lower risk of recurrent ischemic cardiovascular events than those treated with placebo.^57^ The absolute reduction in cardiovascular events in patients (LDL-C concentration 0.65–1.30 mmol/L) with diabetes treated with alirocumab was twice as large as in those without diabetes. Treatment with alirocumab did not increase the risk of new-onset diabetes.^294^ Liao et al.^295^ have reported that in patients with myocardial infarction, lncRNA MIAT participates in the shearing of Wnt7b through targeting miRNA-150-5p and VEGF signaling pathways, and is differentially expressed in patients’ peripheral blood. Therefore, lncRNA MIAT can also be applied as a potential strategy and drug to treat myocardial infarction patients.^295^ Wang et al.^296^ found that cirRNA MFACR was upregulated in myocardial infarction. CirRNA MFACR promoted hypoxia-induced apoptosis of cardiomyocytes by downregulating miRNA-125b. Therefore, targeted inhibition of cirRNA MFACR might serve as a essential target for the treatment of myocardial infarction and the protection of myocardial cells.^296^

#### Heart failure

Some drugs have a crucial function in the treatment of heart failure by regulating noncoding RNA. MRG-110 and CDR132L have been shown to play an important role in the treatment of heart failure by targeting miRNA-92a and miRNA-132, and are currently in phase 1 clinical trials.^297^ In view of cirRNA as a targeted therapeutic target for cardiovascular disease, cirRNA (HRCR) can block the development of cardiac hypertrophy and heart failure, and will be looked forward to becoming a controlling gene for the treatment of heart failure and cardiac hypertrophy in future.^298^ In addition, study demonstrated that ectopic expression of circ-FOXO3 inhibited cell cycle progression by binding to CDK2 and cyclin-dependent kinase inhibitor 1 (or P21), and at the same time, disminished the expression of these proteins in the nucleus and promoted cell aging phenotype. Finally, it provided a new therapeutic strategy for delaying cardiac aging and myocardial protection.^299^

#### Vascular calcification

Noncoding RNAs will be novel therapeutic genes for the treatment of vascular calcification in the future. Zhang et al.^300^ demonstrated that lncRNA-ANCR was an important factor regulating osteoblast differentiation. LncRNA-ANCR might significantly reduce the Runx2 and BMP2 expression and mineralized nodules formation by activating β-GP-induced VSMC autophagy, and weaken the VSMCs osteogenic differentiation, thereby protecting vascular calcification. LncRNA-ANCR could be a pivotal strategy for the treatment of vascular calcification.^300^ Zhu et al.^301^ found that lncRNAs H19 significantly enhanced the Runx2, osteocalcin, ALP, and β‐catenin expression levels in human renal interstitial fibroblasts (hRIFs) by activating the Wnt-β-catenin pathway. It leaded to the formation of mineralized nodules. Finally, lncRNAs H19 accelerated hRIFs osteogenic differentiation and calcification process. Downregulation of XAV939 (Wnt-β-catenin signaling pathway inhibitor) inhibited H19-induced hRIFs osteogenic differentiation, which will serve as a potential drug for vascular calcification future treatment.^301^ In addition, a clinical study showed that exosomes hsa_circRNA_0006859 were upregulated in patients with osteoporosis compared with healthy controls. According to bioinformatics analysis, hsa_circRNA_0006859 might be the sponge of miRNA-431-5p. Hsa_circRNA_0006859 overexpression upregulated the target gene ROCK1 of miRNA-431-5p by inhibiting miRNA-431-5p, significantly decreased the osteocalcin and ALP protein levels in human bone marrow mesenchymal stem cells, and reduced the mineralized nodules formation, thus inhibiting osteoblast differentiation. The results showed that hsa_circRNA_0006859 might be an effective therapeutic gene for preventing vascular calcification.^302^ These studies suggest that RNA therapy is a promising strategy for treating cardiovascular disease (Figs. 5–7). We look forward to applying these findings to clinical disease treatment.

Noncoding RNA plays an important regulatory role in complex life processes and can be considered as a potential therapeutic target for many cardiovascular diseases. Anti-miRNA and antisense oligonucleotides inhibit some specific miRNAs expression in order to regulate the occurrence and development of cardiovascular diseases, which has been used in the cardiovascular diseases clinical treatment. Using the same strategy to regard noncoding RNAs as therapeutic targets in order to improve cardiomyocytes and vessals status and function, which could be a novel approach to treat cardiovascular disease. We hope that in future clinical work, the detection of cardiovascular-related noncoding RNA plasma levels will contribute to determine the disease and severity of patients. It is even hoped to reverse pathological changes in cardiovascular disease and improve the prognosis of patients with cardiovascular disease through gene-targeted therapy.

In Table 5, we report on potential epigenetic therapy drugs for cardiovascular disease. Meanwhile, in Table 6 are included the few most current clinical trials with observational and/or interventional study type for primary and secondary prevention, registered and extracted from the website https://clinicaltrials.gov. These epigenetics-based clinical trials and related epigenetic drug studies are mainly carried out in patients with atherosclerosis, coronary heart disease, heart failure, hypertension, myocardial infarction, and other cardiovascular diseases. However, most researches on the application of epigenetic regulation to cardiovascular disease are still in preclinical trials or early clinical trials stage. Although some epigenetic drugs have not been widely used in clinical practice in cardiovascular diseases. It is believed that through continuous exploration and large-scale clinical research in the future, more emerging epigenetic drugs for the treatment of cardiovascular diseases will be created, in order to better ameliorate the symptoms and prognosis of patients with cardiovascular diseases.

**Table 6:** Clinical Trials With Epigenetics-Based cardiovascular disease Therapeutics

Disease	Study type	Epidrug	Number of participants	Putative epigenetic modification	Recruitment status/phase	NCT number
Atherosclerosis	Observational	Statin	45	DNA/histone methylation Histone methylation HDAC inhibitors	Completed	NCT03354156
Atherosclerosis	Interventional	ALN-PCSSC	501	PCSK9 synthesis	Phase 2	NCT02597127
Atherosclerosis	Observational	–	40	DNA methylation. histone acetylation, noncoding RNA	Completed	NCT02393768
Heart failure	Interventional	Nicotinamide riboside	40	Histone acetylation, noncoding RNA	Early Phase 1	NCT04528004
Heart failure	Interventional	Empagliflozin	105	DNA methylation	Phase 4	NCT03485092
Heart failure	Observational	–	30	miRNAs	Completed	NCT03546062
Heart failure	Interventional	levosimendan	136	miR-660-3p, miR-665 and miR-1285-3p	Phase 4	NCT04950569
Coronary artery disease	Interventional	Incretins	150	Histone acetylation, noncoding RNA	Phase 4	NCT03360981
Coronary artery disease	Interventional	Metformin	68	HDAC activator	Phase 4	NCT02226510
Coronary artery disease	Interventional	Statin	2630	DNA methyltransferases inhibition HDAC inhibitors miRNAs	Phase 4	NCT01715714
Coronary artery disease	Interventional	Rivaroxaban	20	DNA methylation. histone acetylation	Phase 4	NCT05210725
Coronary artery disease	Interventional	Sodium Valproate	122	Histone acetylation	Phase 2	NCT03825250
Coronary heart disease	Interventional	ABT-335, Atorvastatin	682	DNA/Histone methylation HDAC inhibitors	Phase 3	NCT00616772
Coronary heart disease	Observational	–	200	DNA methylation/hydroxymethylation DNMTs and TET family enzyme	Completed	NCT03462277
Hypertension	Interventional	Eplerenone, Amlodipine	300	DNA methylation	Not yet recruiting	NCT04840342
Hypertension	Interventional	Bisphenol A	60	DNA methylation	Completed	NCT02096991
Hypertension	Observational	–	1371	DNA methylation. histone acetylation, noncoding RNA	Recruiting	NCT03002558
Hypertension	Observational	–	600	DNA methylation, acetylation, and histone modifications	Enrolling by invitation	NCT03719703
Takayasu arteritis	Interventional	Leflunomide, Prednisone acetate	116	Noncoding RNA	Unknown	NCT02981979
Myocardial ischemic reperfusion injury	Interventional	–	60	miR-133b; miR-208a	Completed	NCT02149316
Myocardial infarction	Observational	–	1200	miR-126	Completed	NCT01875484
Myocardial infarction	Interventional	Metformin	380	HDAC activator	Phase 3	NCT01217307
Myocardial infarction	Interventional	–	1002	DNA methylation. histone acetylation, noncoding RNA	Completed	NCT00924937
Ischemic heart disease	Interventional	Metformin	100	HDAC activator	Phase 4	NCT01438723
Atrial fibrillation	Observational	Vidaza	14	DNA methylation	Unknown	NCT03298321
Atrial fibrillation	Observational	–	30	DNA methylation	Recruiting	NCT04766814
Cardiopulmonary disease	Observational	–	150	DNA methylation	Recruiting	NCT04859894
Congenital heart defects	Observational	–	100	DNA methylation	Recruiting	NCT04382573
Acute coronary syndrome	Observational	–	100	DNA methylation	Completed	NCT04371809
Pulmonary arterial hypertension	Observational	–	30	DNA methylation	Completed	NCT04282434
Pulmonary hypertension	Interventional	–	96	microRNA and the state of methylation	Recruiting	NCT04224012
Pulmonary arterial hypertension	Interventional	Apabetalone	7	Noncoding RNA	Completed (early phase 1)	NCT03655704
Pulmonary hypertension	Interventional	–	250	microRNA methylation	Recruiting	NCT04224025

## Conclusions

We briefly review the history and mechanisms of epigenetics. At the same time, the mechanisms of epigenetics such as DNA methylation, histone modification, and noncoding RNA in various cardiovascular diseases are comprehensively described. In view of the recent great attention paid to epigenetics in the treatment of cardiovascular diseases, we also summarize the epigenetics drugs related to cardiovascular diseases and all relevant clinical trials. Throughout the current research and application of epigenetics in cardiovascular diseases, it is found that DNA methylation and histone acetylation have been widely and deeply studied in the fields of coronary heart disease, heart failure, and vascular calcification. However, the role of histone methylation in various cardiovascular disease mechanisms remains poorly explored. This can be a new direction worth further excavating in future. In addition, in recent years, more and more studies have confirmed the role of various potential epigenetic drugs in the treatment of cardiovascular disease. At present, in the field of cardiovascular diseases, HDAC inhibitors application in the treatment of atherosclerosis, myocardial infarction, and heart failure has been relatively more studied. The development of HDAC inhibitors has opened up new approaches for the treatment of cardiovascular diseases. However, previous researchers only explored at the animal level. Therefore, by summarizing clinical trials on epigenetic therapy for cardiovascular diseases, we found that DNA methylation and histone acetylation inhibitors have been widely studied in coronary heart disease, myocardial infarction and hypertension, which are currently in phase 1–3 clinical trials. Due to the lack of reliable evidence from large-scale basic and clinical studies, there are still many problems (such as off-target effects) that need to be addressed in the development of noncoding RNA-based therapies. Effective and safe implementation of noncoding RNA therapy into clinical practice remains a major challenge. Large-scale clinical studies are still needed to develop small molecule drugs based on histone methylation and noncoding RNA expression as therapeutic targets.

In summary, epigenetics is a promising field in the diagnosis and intervention of cardiovascular diseases. The rapid development of epigenetics and genomics may serve as a new direction for the precise treatment of cardiovascular diseases. The development of more epigenetics-related drugs with higher specificity, fewer side effects and lower drug resistance for different types of cardiovascular diseases will be the future development goal. The combination of epigenetic regulatory drugs and targeted drugs that drive the regulatory genes of cardiovascular diseases may be an effective target for overcoming drug resistance, which will bring new hope to overcome the difficulties of drug resistance. It is believed that in the near future, the continuous exploration and research, and development of epigenetic regulatory drugs for cardiovascular diseases will have broader application prospects and benefit more patients with cardiovascular diseases. In future, we hope to further explore the molecular mechanism of epigenetics regulating cardiovascular disease, and find more strategies for the prevention and treatment of cardiovascular disease, so as to better guide clinical treatment.
